# Design of an artificial natural killer cell mimicking system to target tumour cells

**DOI:** 10.1177/20417314251349675

**Published:** 2025-09-27

**Authors:** Vaishali Chugh, Vijaya Krishna Kanala, Dagmar Quandt, Suainibhe Kelly, Damien King, Lasse D. Jensen, Jeremy C. Simpson, Abhay Pandit

**Affiliations:** 1 CÚRAM, SFI Research Centre for Medical Devices, University of Galway, Ireland; 2 Cell Screening Laboratory, UCD School of Biology and Environmental Science, University College Dublin, Belfield, Dublin 4, Ireland; 3 Fraunhofer Project Centre for Embedded BioAnalytical Systems, Dublin City University, Dublin 9, Ireland; 4 Department of Health, Medicine and Caring Sciences, Linköping University, Sweden

**Keywords:** NK cell membrane, gelatin microspheres, biomaterials, 3D spheroids, zebrafish xenograft breast tumour model

## Abstract

NK cell mimics are assemblies of a cell membrane and a template that replicate biomimetic features and physicochemical properties, respectively. To develop this targeted drug delivery system, gelatin microspheres (cG) were fabricated using a water-in-oil emulsion and reinforced via DMTMM cross-linking to exhibit tunable Young’s modulus, a critical parameter for cell–material interactions. These microspheres were subsequently coated with membranes derived from the human NK cell line KHYG-1 to form biomimetic NK cell mimics (cGCM), combining physicochemical control with bioinspired functionality. These engineered cGCM were non-toxic, non-inflammatory, and capable of reducing macrophage uptake by ~10% when incubated with differentiated THP-1 cells. In vitro studies demonstrated significant interaction/ proximity of the cGCM with cancer cells in 2D cultures of breast cancer cells (MDA-MB-231), 3D spheroids of liver (HepG2), and colon (HT-29) cancer cell models, and a zebrafish breast cancer xenograft (MDA-MB-231) model. The cGCM also evaded macrophage detection in a Kdrl:EGFP Spil:Ds Red zebrafish model. Furthermore, in a pilot assessment, loading and release of the sialyltransferase inhibitor (STI, 3Fax-Peracetyl Neu5Ac) using cGCM significantly reduced α-2,6 sialylation in 2D cultures of MDA-MB-231 cells, demonstrating the STI’s intact functionality in inhibiting sialylation. By integrating bioinspired membranes with mechanically tunable gelatin-based carriers, our system demonstrates a multifunctional immune-mimicking platform with relevance to tissue engineering, tumour modelling, immune modulation, and drug delivery. These findings offer a promising foundation for future therapeutic strategies in cancer research and immuno-engineering.

## Introduction

Designing delivery systems that can effectively interact with their physiological micro-immune-environment and conserve functionality is a vastly unmet need in the field of biomedicine. Non-specific absorption of blood proteins on their surface and elimination by the mononuclear phagocyte system (MPS) are example scenarios that impede their bio-interfacing.^1–3^ Cell membrane-coated (CMC) mimics have emerged as a reliable alternatives to these traditional counterparts owing to their ability to integrate biocompatibility, stealth properties, active targeting, and site accumulation behaviour of cell membranes onto templates with tunable physiochemical properties.^4–7^ Additionally, they eliminate the need for complex chemical processing and traditional synthetic modifications. Numerous cell types, including red blood cells,^8–10^ platelet,^11–13^ immune cells,^14,15^ and cancer cells,^16–19^ have been utilized for designing CMC mimics for various therapeutic applications. Mimics with immune cell membranes such as macrophages,^20–24^ neutrophils,^15,25^ and T-cells^26^ have shown promising results in cancer therapy, due to the presence of tumour-targeting receptors on their surface.

While red blood cell (RBC) membranes are widely used for their excellent immune evasion capabilities due to their innate “self” markers (e.g. CD47), they lack active targeting properties, limiting their effectiveness in tumour-specific delivery. Macrophage and neutrophil membrane-coated systems offer improved tumour-homing capabilities due to inflammation-resolving functions, but may also carry immunosuppressive signals or promote pro-tumour effects depending on their polarization states. T-cell membranes, although offering strong antigen-specific targeting via T-cell receptors, require prior activation and can vary in functional stability, complicating reproducibility. In contrast, NK cell membranes provide an optimal balance by combining inherent tumour recognition (via receptors like NKp30 and NKG2D), consistent anti-tumour identity, and immune evasion without prior activation. These unique attributes position NK cell membrane-coated systems as a highly promising and versatile platform for tumour-targeted therapies.

KHYG-1 is a human NK cell line closely resembling primary NK cells, characterized by high levels of activating (DNAM-1, NKp30, NKG2D), and adhesion (CD11a), and NK identifying (CD56) surface receptors. These cells does not require prior activation (like primary NK cells) and the scalability of this cell line enables harvesting sufficient amounts of cell membrane, therefore was selected for designing NK cell mimics.^27–31^

Structural templates play a crucial role in shaping the mechanical properties of CMC mimics. Elasticity in the drug delivery systems provides additional mechanical characteristics that enhances circulation time, bio stability, cellular uptake, tumour penetration, immune evasion, biocompatibility, and mechanical adaptability. Although, for nanoparticles smaller than 200 nm, flexibility might be a less critical factor but for microparticles (~5 µm), elasticity becomes a key factor, particularly in cancer therapy. While the significance of elasticity has been well established in scaffolds and cell culture substrates,^38–40^ but has been partially explored in drug delivery systems,^35–37^ its full potential remains largely unexamined. Recent advances have highlighted the advantages of tunable mechanical properties in improving particle performance in vivo. For instance, studies on RBC-mimicking microparticles have shown that particles with tunable elasticity (10–63.8 kPa) exhibit improved circulation and biodistribution.^36,37^ While our system does not aim to mimic RBC function directly, these findings highlight the broader importance of mechanical adaptability in enhancing therapeutic performance. Building on this, we explored gelatin—a structurally tunable and biocompatible biomaterial widely used in tissue engineering—as the core material for constructing NK cell mimics. The elasticity of gelatin microspheres is influenced by bloom strength, concentration, cross-linking degree, and temperature. To enhance gelation and mechanical stability, we selected high-bloom (300) Gelatin A. Additionally, Gelatin A’s isoionic range (6.5–9) facilitates amide bond formation, allowing precise modulation of mechanical properties through DMTMM cross-linking, making it an optimal template for NK cell mimic design.^41,42^ Hence, the overarching goal of the study is to replicate NK cell surface characteristics, size, and relevant cell-like mechanical properties.

**Scheme 1.**
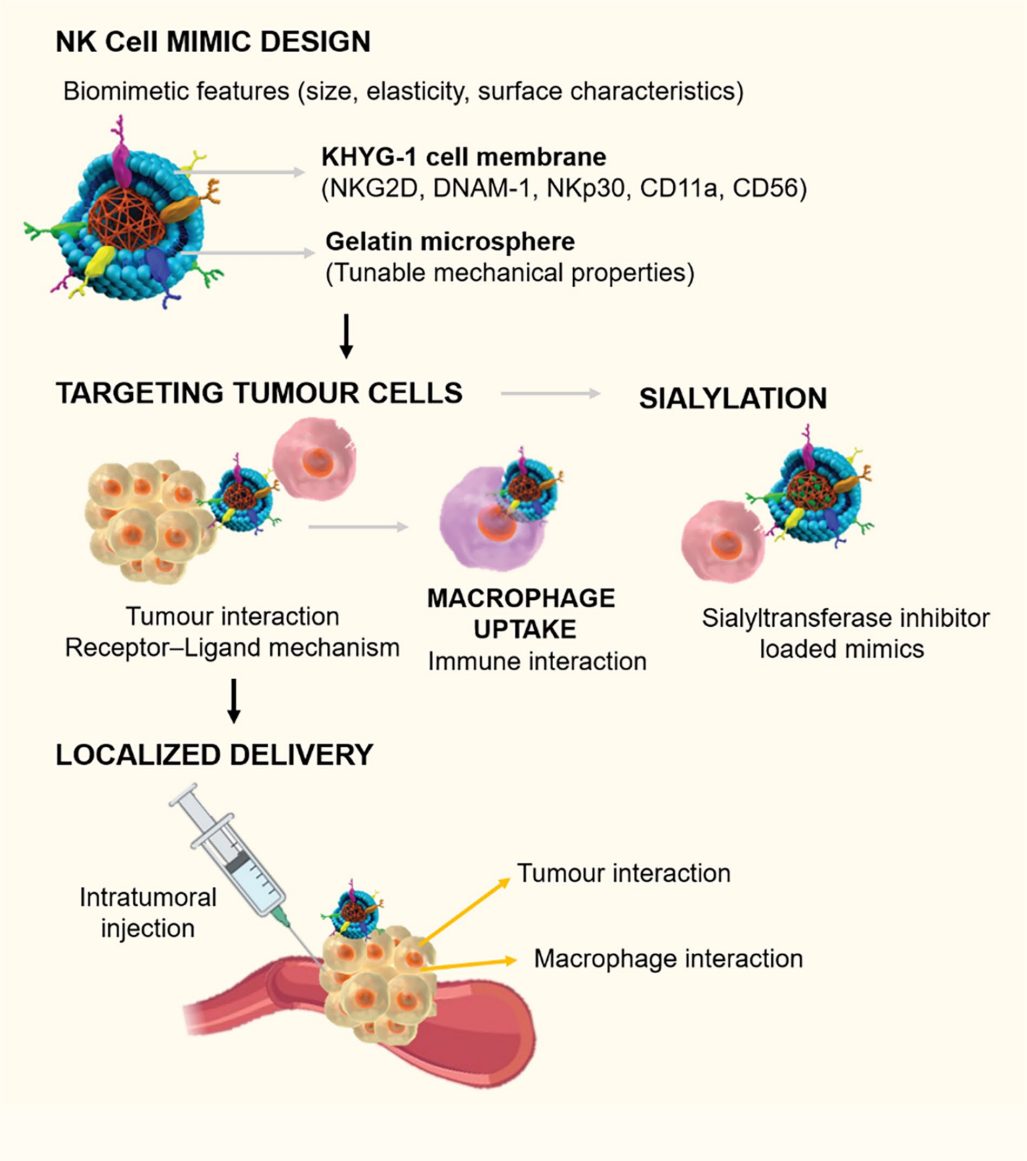
Overview on key conceptual elements of the study, including biomimetic design, tumour targeting capability, interaction with macrophages, and the localized delivery strategy.

**Scheme 2.**
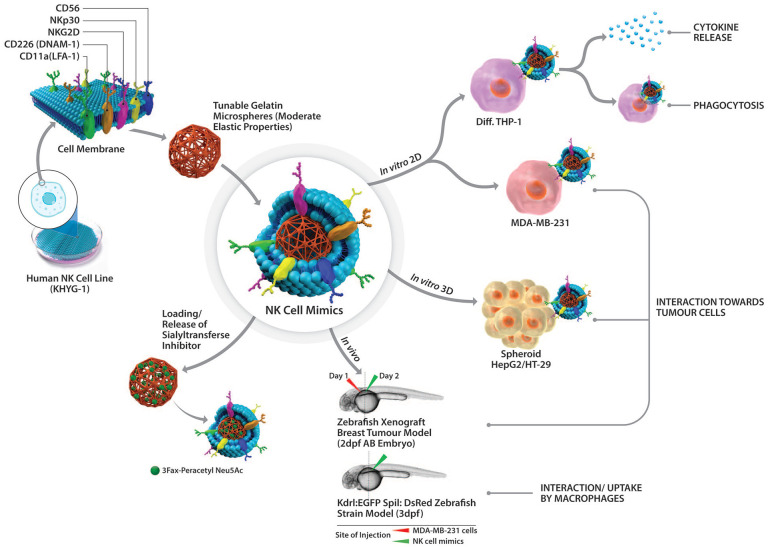
Overview of NK cell mimics’ comprehensive study: design, interactions and therapeutic potential in tumour therapy. NK cell mimics were designed by coating KHYG-1 cell membrane onto gelatin microspheres, exhibiting moderate elasticity. NK cell mimics’ interaction with macrophages (2D in vitro differentiated THP-1 model) were investigated to examine their pro-inflammatory response and phagocytosis. The NK cell mimics’ interaction towards tumour cells without prior activation were evaluated in various in vitro models using 2D breast cancer cell cultures (MDA-MB-231), 3D spheroids of liver (HepG2), and colon (HT-29) cancer cells. Further, their interaction with breast cancer cells and macrophages in an in vivo zebrafish model was investigated. Finally, NK cell mimics’ loading and drug release behaviour was assessed using sialyltransferase inhibitor (STI, 3Fax-Peracetyl Neu5Ac) as a relevant model drug.

In this study (Schemes 1 and 2), we report assembly of NK cell mimics by coating KHYG-1 cell membrane onto gelatin microspheres and their physiochemical and biological characterization. By exploiting the tunable mechanical properties of gelatin, we create microparticles using DMTMM (4-(4,6-dimethoxy-1,3,5-triazin-2-yl)-4-methylmorpholinium) as a cross-linker in a water-in-oil emulsion that exhibit moderate elasticity, emulating some cell-like physicochemical characteristics. DMTMM is a non-toxic, water soluble, zero-length cross-linker, widely used in both chemical and biochemical scenarios.^32,33^ We determined the mechanical properties of gelatin microspheres using nano-indentation and lab-on-disc centrifugal microfluidics. Additionally, the interaction of NK cell mimics with macrophages was investigated to determine the inflammatory response and its ability to evade from macrophage detection. The interaction of the mimics towards cancer cells was examined using 2D breast cancer cell cultures (MDA-MB-231), 3D spheroids of liver (HepG2) and colon (HT-29) cancer cells. For in vivo studies, we opt for a larval zebrafish model. Zebrafish larvae are transparent and have a human-like innate immune system which include macrophages but have not yet developed adaptive immunity (e.g. T- and NK-cells) that could interfere with the investigations. This allows accurate, high-resolution visualization of tumour cell-microsphere and host macrophage-microsphere interactions required to understand the differences in therapeutic potential and clearance of the microspheres.^34–40^ No other model system can provide the sub-cellular resolution of these critical interactions in vivo. Zebrafish larvae are furthermore a popular and well-characterized model system for cancer studies^41–47^ and have been used extensively in the past by our and other labs for studies on microsphere pharmacology and biology.^48^ Due to these qualities and validated relevance of zebrafish larvae for studying microsphere biology, this platform was chosen for evaluation of the NK-cell mimic in this study.

To assess the effectiveness of our NK cell mimics in releasing therapeutic payloads, we conducted a pilot study focussing on altered glycans, particularly through the use of sialyltransferase inhibitors (STIs).^49^ Sialyltransferases are enzymes responsible for transferring sialic acid to sugar chains on glycoproteins and glycolipids, a process that plays a critical role in cell-cell interactions and immune evasion.^50^ Overactive sialyltransferases can lead to hypersialylation, which is often associated with cancer progression and metastasis due to its role in masking cancer cell antigens and reducing immune recognition.^51,52^ Therefore, inhibiting sialyltransferases has emerged as a promising strategy to counteract hypersialylation and enhance the immune system’s ability to target and destroy cancer cells. This approach has been the focus of numerous studies exploring effective STIs as potential therapeutic agents.^49,53^ In our study, we specifically investigated 3Fax-Peracetyl Neu5Ac, a well-regarded sialic acid analogue.^54–56^ This compound was chosen for its potential to interfere with the overactivity of sialyltransferases and its ability to affect glycan modifications in cancer cells. We evaluated the loading and release pattern of 3Fax-Peracetyl Neu5Ac from NK cell mimics and tested its function in the MDA-MB-231 breast cancer. This approach was designed to ensure that the STI remained effective and intact during the loading and release processes, providing insight into its potential for targeted cancer therapy. By integrating both in vitro and in vivo methodologies, our study aims to validate the use of NK cell mimics and STIs as innovative tools for cancer treatment, offering valuable insights into their practical applications in targeted tumour therapies.

## Materials and methods

### Materials

Gelatin (porcine skin, gel strength ~300 g Bloom, Type A), 4-(4,6-dimethoxy-1,3,5-triazin-2-yl)-4-methyl-morpholinium chloride (DMTMM), mineral oil, sorbitan monooleate 80, Dextran-FITC (MW: 40,000), radioimmune precipitation (RIPA) buffer, phenylmethylsulfonylfluoride (PMSF), phorbol 12-myristate 13-acetate (PMA), 3Fax-Peracetyl Neu5Ac (sialyltransferase inhibitor), RPMI 1640 medium with L-glutamine and sodium bicarbonate, foetal bovine serum (FBS), 1 mM sodium pyruvate solution, 1% Penicillin-streptomycin (Pen-Strep), tricane, 0.01% Poly-L-Lysine (PLL) solution, 2,4,6-Trinitrobenzenesulfonic acid (TNBS) solution, NaCl, and CaCl_2_ were purchased from Sigma-Aldrich. Human IL-2 IS, research grade and CD45_APC_Cy7 dye were purchased from Miltenyi Biotech. Dextran cascade blue, Pierce™ BCA Protein Assay Kit, X-ray films (CLXPosure™ Film), *Hoechst 33342* fluorescent dye and Vybrant™ DiI were purchased from Thermo Fischer Scientific. CM-DiI cell tracker dye was purchased from Biosciences. Human interleukin 1β (IL-1β, DusoSet) and human tumour necrosis factor α (TNF-α, DuoSet) were purchased from R&D Systems. Protease inhibitor cocktail (cOmplete™, Ethylenediaminetetraacetic acid (EDTA)-free), and phosphatase inhibitor cocktail (PhosSTOP™) were purchased from Roche. Corning^®^ 96-well flat clear bottom black polystyrene TC-treated microplates was purchased from Life Sciences. Most of the primary antibodies (anti-NKp30, anti-CD226, anti-CD11a, Anti-NKG2D) were purchased from Abcam. Anti-CD56 primary antibody and all the secondary antibodies (HRP-goat anti-rabbit and HRP-goat anti-mouse) were purchased from ThermoFischer Scientific. Borosilicate glass needles were purchased from World Precision. Microloader™-microcapillary tips were purchased from Eppendorf. 1-Phenyl-2-Thiourea (PTU) was purchased from Thermo Scientific Alfa Aesar.

### Breeding and maintenance of zebrafish

Zebrafish embryos from the AB strain and the Kdrl:EGFP Spil:Ds Red strain were bred and maintained at the zebrafish facility in Linköping, Sweden. These zebrafish were used for all in vivo experimental procedures. The zebrafishes were fertilized and stored as reported previously.^57,58^ Briefly, after successful breeding, fertilized zebrafish eggs were collected and placed in Petri dishes. These eggs were then incubated at 28°C in E3 embryo medium with a pH of 7.2. The composition of the E3 embryo medium per litre of purified water included 0.29 g of NaCl, 0.082 g of MgSO_4_, 0.048 g of CaCl_2_, and 0.013 g of KCl. Additionally, 0.2 mM of PTU was supplemented in the E3 embryo medium to prevent pigmentation in the developing larvae. The animal experiments using zebrafish larvae was conducted before their independent feeding stage (around 5 days post-fertilization), which is exempt from ethical approval under EU regulations.

### KHYG-1 cell culture conditions

KHYG-1 cells were cultured at cell density of 300,000 to 500,000 cells/ml in RPMI 1640 medium with L-glutamine and sodium bicarbonate supplemented with 10% heat inactivated foetal bovine serum, 1 mM sodium pyruvate solution, 1% Penicillin-, and 10 ng/ml human IL-2 IS research grade.

### Isolation of KHYG-1 cell membranes

About 300 million KHYG-1 cells were homogenized by using the combination of hypotonic buffer (10 mM HEPES, 42 mM KCl, 5 mM MgCl_2_, pH 7.3) and physical disruption technique (~200 strokes of dounce homogenizer). Further, cell membrane was isolated using discontinuous sucrose gradient ultracentrifugation method. Briefly, sucrose gradients were prepared using 30%, 40%, and 55% sucrose solution in 0.9% NaCl solution. On top of the gradient added homogenized cell solution and centrifuged at 28,000*g* for 1 h in Optima™ XL-100 K ultracentrifuge at 4°C. The interface between 30% and 40% was collected and centrifuged at 100,000*g* in Optima™ XL-100 K ultracentrifuge at 4°C. After the final centrifugation, discarded the supernatant and collected the cell pellet. The final cell pellet obtained was the KHYG-1 cell membrane.

### Synthesis of cross-linked gelatin microspheres

Gelatin microspheres were obtained using water-in-oil emulsion technique.^59^ An aqueous gelatin solution of 10% w/w (1 ml) was added drop by drop in mixture of mineral oil (4.2 ml) and sorbitan monooleate 80 (42.46 μl, final concentration of 1.0% w/v) in round bottom flask involving heating at 55°C in a ceramic hot plate with mechanical agitation at 2000 rpm. The resulting emulsion was maintained under this condition for 15 min and then cooled at room temperature using water bath and 2000 rpm agitation. Further, the emulsion was cooled down between 10°C and 15°C with an ice bath while stirring. After 15 min, 40 ml of cold acetone was added dropwise to the emulsion and the mixture was stirred for another 15 min at 2000 rpm. Then, the whole solution with the spheres was transferred to 50 ml falcon tube to be centrifuged at 3400 rpm for 2 min at room temperature. The supernatant was discarded and acetone was again added to the solid residue to wash away the mineral oil left in the sample. The tube was centrifuged again at 3400 rpm for 2 min and the washing step was repeated three times. Further, gelatin microspheres were cross-linked using zero-length cross-linker that is, DMTMM. 2 ml of crosslinking medium was prepared using the 7:3 v/v ratio of acetone: 0.01 M sodium hydroxide for 100 mg of microspheres. 50 mM DMTMM was used for cross-linking the microspheres. The cross-linking medium was divided into two equal portions. About 1 ml of the medium was used to transfer the 100 mg of microspheres in the round bottom flask and agitated at 2000 rpm. Rest of the 1 ml of medium was used to dissolve the DMTMM and added in the round bottom flask with the microspheres on agitation. The whole mixture was kept for 3 h at room temperature at 2000 rpm. After 3 h, the mixture was transferred into 15 ml falcon tube and centrifuged at 3400 rpm. Further, the supernatant was discarded and 10 ml of acetone was added and centrifuged again. This washing step with acetone was repeated thrice to wash away the unreacted DMTMM. The cross-linked microspheres were kept in open falcon tube to allow air drying for at least 12 h. For preparing dextran-FITC or dextran cascade blue loaded gelatin microsphere, 0.05 mg of dextran-FITC or cascade blue was dissolved with 1 ml of gelatin aqueous solution in the starting step and further all the steps mentioned above were the same. The aluminium foil was used to cover the reaction to avoid bleaching of FITC or cascade blue.

### Mechanical properties (Young’s modulus) using nano-indentation

Three different cross-linked gelatin hydrogels were prepared in the same manner as gelatin microspheres using 10 mM DMTMM, 50 mM DMTMM and 100 mM DMTMM. Two 500 μl 10% aqueous gelatin solution was dropped on a silicon mould placed on ice to provide structure to the hydrogel for each set of DMTMM concentration. After few minutes, these two hydrogels were dipped in 2 ml of specific DMTMM solution aqueous solution each and kept on shaker for 3 h for crosslinking at room temperature. Further, after 3 h of crosslinking the hydrogels were placed on glass cover slip in 24 well plate and topped up with 500 μl of deionized water for the measurement in Optics11Life-Pavone High-Throughput Indentation Platform. For measuring elastic modulus or Young’s modulus of the three different cross-linked gelatin hydrogels (three replicates each), displacement control mode was operated with the same parameters that is, (1) tip radius of the probe = 9.50 μm, (2) stiffness of the probe = 0.200 N/m, (3) calibration factor = 2.072, (4) air temperature = 34.450°C, (5) humidity = 4.350%, (6) Poisson’s ratio = 0.5. Ten measurement/indentations were made for each sample covering the whole area (including centre and corners) of sample as much as possible. During the mode of operation, the instrument recorded piezo movement (blue curve) and cantilever bending (green curve) and the validation of these results was solidified through Load-indentation curves, where the Hertz fit (red curve) perfectly overlapped with the piezo movement (blue curve), which ensured the accuracy of the young’s modulus assessment for all hydrogel types (Figure S1). Finally, average of all the calculated young’s modulus by the in-built DataViewer software version (V1.6.0) with the instrument was plotted with standard deviation for each sample.

### Lab-on-disc centrifugal microfluidics

**Figure 1.**
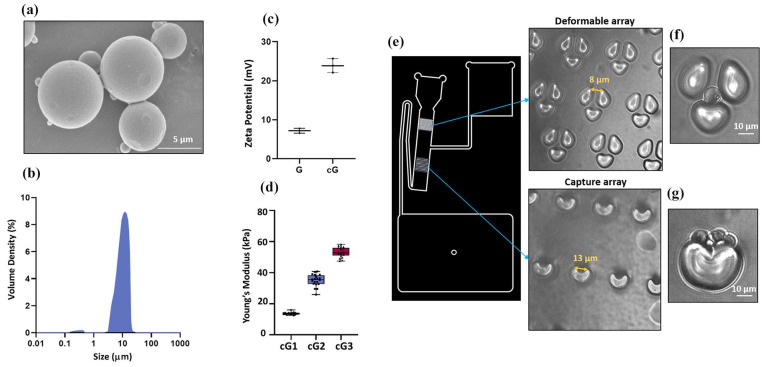
Characterization of gelatin microspheres: (a) field emission scanning electron microscopic (FESEM) image cross-linked gelatin microspheres (cG) using 50 mM DMTMM, scale bar = 5 μm, (b) size distribution of cG by laser diffraction, (c) surface charge on gelatin microspheres before crosslinking (G) and after crosslinking (cG) using zetasizer (*N* = 3), Error bars represent standard deviations, (d) Young’s modulus estimation of gelatin hydrogels cross-linked with 10, 50, and 100 mM DMTMM, termed as cG1, cG2, cG2 respectively, *N* = 3, Error bars represent standard deviations, (e) design of microfluidics chip, consists of deformability array with gap sizing of 8 μm, and capture array with v-cup sizing of 13 μm, (f) representation of deformable gelatin microspheres (>8 μm) captured in the deformable array, scale bar = 10 μm, and (g) representation of aggregated spheres/smaller spheres/non-deformable spheres captured in V-cups, scale bar = 10 μm. Graphs are plotted in box and whiskers format with max and min value showing all data points.

The microfluidics chip design was inspired by a previously established concept with some modifications.^60–62^ Briefly, Figure S8 wafers were first prepared using PDMS and then, these wafers were casted, degassed, oven cured, cut and then bonded to the glass side. Further, the bonded chips were vacuumed for 30 min and chips were primed with buffer using degas driven flow. After the formation of chips with deformability array of gap size 8 μm and V-cup size in capture array of 13 μm, the gelatin microspheres were loaded using pipette. Finally, the loaded chips were mounted on 3D printed disc on spin stand to spin at 10 Hz for 40 min. The spheres transferred through the chip under sedimentation conditions and pass through deformability and capture array. Following the centrifugation process, the percentage of deformable gelatin microspheres present in the provided sample was calculated by analysing the number of microspheres larger than 8 μm trapped in the deformability array and the microspheres (aggregated/non-deformable/small sized) in the capture array. Some of the representative images of the chips and set up of the lab-on-disc centrifugal microfluidics has been presented in Figures 1(e) and S2.

### Fabrication of NK cell mimics

About 1 mg of the gelatin cross-linked microspheres and 1 mg of isolated cell membrane were dispersed in 1 ml of deionized water in separate two capped 2 ml centrifuge tube and sonicated at 100 W for 5 min on ice in water bath sonicator. After 5 min, 1 mg/ml solution of gelatin microspheres was mixed with 1 mg/ml isolated cell membrane solution and sonicated at 100 W for 5 min on ice in water bath sonicator. Further, the mixture was centrifuged at 14,000 rpm in pico™ 17 microcentrifuge at 4°C for 20 min.

### Protein loading yield

Protein loading yield is defined as the weight ratio of immobilized proteins to the gelatin microspheres after cell membrane coating.^63–65^ About 1 mg of gelatin microspheres and NK cell mimics were added to 100 μl of cold radioimmune precipitation (RIPA) buffer (50 mM TrisHCl, pH 8.0, 150 mM NaCl, 0.02% sodium azide, 0.1% sodium dodecyl sulphate (SDS), 1% Nonidet P-40, 0.5% sodium deoxycholate) with protease inhibitor cocktail (1:100), phosphatase inhibitor cocktail (1:10) and phenylmethylsulfonylfluoride (1:50). After 15 min on ice, vortexed and centrifuged the sample at 14,000 rpm in pico™ 17 microcentrifuge at 4°C for 20 min. Supernatant after the centrifugation was used for protein quantification. Total protein concentration on gelatin microspheres and NK cell mimics was determined by Pierce™ BCA Protein Assay Kit to measure the absorbance at 562 nm. Bovine serum albumin (BSA) protein was used for the standard curve. Each set of sample treatment was done in triplicates.

(1) $$\mathrm{Protein}\;\mathrm{loading}\;\mathrm{Yield}\;\left(\%\right)=\;\frac{\mathrm{Protein}\;\mathrm{quantified}\;\mathrm{per}\;1\;\mathrm{mg}\;\mathrm{of}\;\mathrm{NK}\;\mathrm{cell}\;\mathrm{mimics}}{\mathrm{Protein}\;\mathrm{quantified}\;\mathrm{per}\;1\;\mathrm{mg}\;\mathrm{of}\;\mathrm{isolated}\;\mathrm{cell}\;\mathrm{membrane}}*100$$

### Protein absorption study

About 1 mg each of gelatin microspheres and NK cell mimics were incubated in 500 ml of foetal bovine serum for 3 h at 37°C with constant agitation. Further, gelatin microspheres and NK cell mimics were separated by centrifugation at 14,000 rpm in pico™ 17 microcentrifuge for 20 min. Both the spheres’ pellets were washed three times in Dulbecco’s phosphate-buffered saline buffer, centrifuged and discarded the supernatant. After the washing steps, the pellets were added to 100 μl of cold radioimmune precipitation (RIPA) buffer (50 mM TrisHCl, pH 8.0, 150 mM NaCl, 0.02% sodium azide, 0.1% sodium dodecyl sulphate (SDS), 1% Nonidet P-40, 0.5% sodium deoxycholate) with protease inhibitor cocktail (1:100), phosphatase inhibitor cocktail (1:10) and phenylmethylsulfonylfluoride (1:50). After 15 min on ice, vortexed and centrifuged the sample at 14,000 rpm in pico™ 17 microcentrifuge at 4°C for 20 min. Supernatant after the centrifugation was used for protein quantification. Total protein concentration on gelatin microspheres and NK cell mimics was determined by Pierce™ BCA Protein Assay Kit to measure the absorbance at 562 nm. Bovine serum albumin protein was used for the standard curve. Each set of sample treatment was done in triplicates.

### Protein analysis of the NK cell mimics

The protein profile in the gelatin microspheres, isolated NK cell membrane, and NK cell mimics was visualized, analysed, and compared qualitatively by loading and running the same amount of protein in a 12% of SDS-PAGE gel that allowed the separation of protein-based on mass. After the separation of proteins, these gels were stained with irreversible 0.1% Coomassie brilliant blue G-250 dye (w/v in 50% methanol + 5% acetic acid glacial) for 20 min with gentle agitation. Further, the gel was de-stained using de-staining solution (20% methanol + 5% acetic acid glacial) under shaking, to release all the unbound coomassie brilliant blue dye. The de-staining solution was replenished several times until background of the gel is fully de-stained. Finally, de-stained gel was scanned on gel-scanner.

### Protein detection in the NK cell mimics

The total protein was extracted from KHYG-1 cells, isolated cell membrane and NK cell mimics using ice cold RIPA buffer and quantified the total protein content using BCA assay kit as mentioned in the above sections. Further, 5 µg protein from each sample (KHYG-1 cells, isolated cell membrane and NK cell mimics) was separated in a desired SDS polyacrylamide gel electrophoresis (10% or 12% gel depending on specific antibody, mentioned in Table S1) and transferred to a Hybond^®^ ECL™ nitrocellulose membrane. Membranes were stained with Ponceau S to visualize successful transfer and protein loading. The membrane was blocked with 5% milk depending upon the primary antibodies’ suitability to avoid non-specific binding. This was followed by specific primary antibody incubation overnight at 4°C and dilution as mentioned in Table S1. All washing steps were carried out in Tween-20 in TBS (0.1%). Next, horseradish peroxidase-conjugated secondary goat anti-rabbit or goat anti-mouse antibodies (prepared in 5% milk with dilutions mentioned in Table S1) were applied, followed by enhanced chemiluminescence detection. Signals from protein bands were captured on X-ray films.

### Visualization of NK cell membrane onto gelatin microspheres

Three imaging techniques including transmission electron microscopy (TEM), field-emission scanning electron microscopy (FESEM), and confocal laser scanning microscopy (CLSM), were used to examine the structural integrity and morphology of the NK cell mimics. First, prepared 1 mg/ml NK cell mimics sample in deionized water. For TEM, added 10 μl into copper TEM grids. Left to dry for 24 h and imaged the sample in Hitachi 7500 (B-076 HBB). For FESEM, added 20 μl of NK cell mimics on an aluminium foil piece on carbon stab. The sample was sputter coated for 3 min at 2.2 kV and imaged using scanning Electron Microscope Hitachi S-4700 EDX (B-076 HBB). For CLSM, NK cell mimics were using 1 mg of dextran-cascade blue loaded gelatin microspheres and 1 mg of wheat germ agglutinin (WGA)-FITC labelled cell membrane. Appropriate (~20 μl) amount of NK cell mimics were placed on glass slide, covered with coverslip and let it dry for few min and applied transparent paint on the side of cover slips to avoid movement of coverslip while imaging. The glass slide with sample is covered with aluminium foil to avoid bleaching of the dyes. Then, the fluorescent colocalization was observed in Olympus^®^ FV1000 Confocal Laser Scanning Microscope with Olympus FLUOVIEW™ Ver.4.2b software.

### *In vitro* cytokine release assay

The pro-inflammatory behaviour of the prepared gelatin microspheres (cG) and NK cell mimics (cGCM) was tested using standard cytokine release assay (i.e. enzyme-linked immunosorbent assay (ELISA)). In brief, 50,000 THP-1 cells were seeded in 24 well plate and differentiated using 100 ng/ml phorbol 12-myristate 13-acetate (PMA) for 24 h. After PMA treatment, THP-1 cells adhered to the surface of the well plate. Further, removed the old medium gently and washed with Dulbecco’s phosphate-buffered saline (DPBS) twice. Further, differentiated THP-1 cells were treated with gelatin microspheres (cG, 500 μg/ml), NK cell membrane (CM, 500 μg/ml) and NK cell mimics (cGCM, 500 μg/ml) at 37°C for 24 h. After 24 h incubation, cell culture supernatant were collected, centrifuged, removed debris and stored at −20°C as small aliquots. For cytokine assay, samples were thawed and analysed for pro-inflammatory cytokines, human interleukin 1β and human tumour necrosis factor α using ELISA and as per the manufacture recommendations. For positive control, cells were dosed with 100 ng/ml of lipopolysaccharide for 3 h. Each set of sample treatment was done in triplicates.

### *In vitro* cellular uptake studies using differentiated THP-1 cells

First, 1 × 10^6^ THP-1 cells were seeded in petri dish and differentiated using 100 ng/ml PMA for 24 h at 37°C to convert it into activated macrophages. After PMA treatment, THP-1 cells adhered to the surface of the petri dish. Further, removed the old medium gently and washed with Dulbecco’s phosphate-buffered saline (DPBS) twice. About 1 mg of dextran-cascade blue loaded gelatin microspheres (cG) and dextran-cascade blue loaded NK cell mimics (cGCM) were re-suspended in 1 ml serum free media (RPMI 1649 media supplemented with 1% Penicillin-Streptomycin) and sonicated in water bath sonicator for 5 min before adding it on the cells. Approximately, 1 mg of spheres have around 1 million particles according to haemocytometer calculations. The differentiated THP-1 cells were treated with 1 mg of dextran-cascade blue loaded gelatin microspheres (cG) and dextran-cascade blue loaded NK cell (cGCM) mimics prepared in THP-1 cell culture media without FBS for 3 h at 37°C. After 3 h, the unreacted gelatin microspheres and NK cell mimics with the old media was removed and washed the cells with DPBS twice. Further, the cells were detached using trypsin-EDTA for 4 min at 37°C and scratching the whole dish using cell scratcher one time gently. The detached cells were topped up with culture media, centrifuged to collect the cell pellet with up taken spheres and washed with DPBS twice. In the last step, the THP-1 cells were labelled with CD45_APC_Cy7 dye. The uptake studies were analysed using a 1:1 cell to spheres ratio, 3 h at 37°C. Alexa Flour™ 488 tagged Zymosan A (S. cerevisiae) bioparticles™ was used as positive control and temp 4°C was used as a negative control. The individual cells were imaged and analysed the uptake of micropspheres using **ImageStream**^®^ **X Mark II** Imaging Flow Cytometer.

### *In vitro* interaction studies with breast cancer cells (MDA-MB-231)

MDA-MB-231 cells were first stained with CM-DiI cell tracker dye and seeded 20,000 cells/well of Corning^®^ 96-well flat clear bottom black polystyrene TC-treated microplates. After 24 h, removed the old medium and washed with Dulbecco’s phosphate-buffered saline (DPBS) twice. About 1 mg of dextran-FITC loaded gelatin microspheres (cG) and NK cell mimics (cGCM) were re-suspended in 1 ml serum free cell culture medium (RPMI 1649 media supplemented with 1% Penicillin-Streptomycin) and sonicated in water bath sonicator for 5 min before adding it on the cells. Approximately, 1 mg of spheres have around 1 million particles according to haemocytometer calculations. Accordingly, different cell: spheres ratio (1:1, 1:5, 1:10 and 1:20) was added to the cells in the each well for 24 h at 37°C. Every set of sphere treatment was done in triplicates. After 24 h, the old medium was removed and washed with DPBS thrice to remove any unreacted cG and cGCM with the cells followed by fixation of the cells using 4% paraformaldehyde (PFA) solution for 15 min. Finally, the nuclei of the MDA-MB-231 was stained with *Hoechst 33342* fluorescent dye using 1:1000 dilution for 10 min and washed with DPBS twice. Further, the cells were topped up with 50 μl DPBS and were imaged in Operetta^®^ High Content Imaging System (PerkinElmer) and number of FITC-labelled spheres interacted with the MDA-MB-231 cells were determined using in-built harmony 4.8 software installed with operetta instrument. The data obtained was normalized with the highest number of spheres interaction with 1:20 cell: sphere ratio (saturated ratio). Every set of sphere treatment with the MDA-MB-231 cells was done in triplicates.

### *In vitro* interaction studies with 3D spheroids of liver (HepG2) and colon (HT-29) cancer cells

The 3D spheroids of HepG2 and HT-29 cells was prepared in CellCarrier Ultra 96-well microplates (Perkin Elmer) as described in an established protocol.^66^ Briefly, the wells were first coated with matrigel (15 μl) basement membrane matrix phenol red-free (Corning) that was thawed on ice overnight before use. The microplate was placed on a pre-chilled metal surface on ice during all preparation steps. Matrigel mixture (4 mg/ml) was prepared by diluting matrigel with phenol red-free ice cold McCoy’s media. The 96-well plate was centrifuged at 4°C, 900 rpm for 15 min to allow uniform distribution of the matrigel layer, followed by incubation for 30 min at 37°C to allow gelation of matrigel matrix to occur. Approximately 3000 HT-29 or HepG2 cells were dispensed in phenol red-free complete medium to the matrigel bed, and after 1 h of incubation at 37°C the complete growth medium in the overlay was supplied with matrigel to give a 2% (w/v) concentration in a final 60 μl volume. On the second day of 3D culture, the complete growth medium was changed as before. Further, the HT-29 or HepG2 spheroids were treated with dextran FITC-loaded gelatin microspheres (cG) and dextran FITC-loaded, CM-DiI cell membrane labelled NK cell mimics (cGCM). First, 1 mg of cG and cGCM were suspended in 1 ml serum free medium and sonicated for 10 min in a water bath sonicator. Approximately, 1 mg of spheres have around 1 million particles according to haemocytometer calculations. Accordingly, the cG and cGCM were incubated with the HT-29 and HepG2 spheroids with different cell: spheres ratio (1:1, 1:5, 1:10, 1:20) for 3 h. After 3 h, the cells were washed first with complete medium and re-added the complete medium. Further, kept the cells with the spheres for 24 h at 37°C. After 24 h, the medium was removed from the cells, they were washed with PBS and further, fixed with 4% paraformaldehyde (PFA) solution. After fixation, cells were further washed with PBS and the nuclei were stained with *Hoechst 33342* fluorescent dye at final concentration of 1 μg/ml. The stained cells were washed again with PBS and held in PBS for imaging. The imaging was done in an Opera Phenix High Content Screening System (Perkin Elmer) and criteria to classify nuclei region, small and large spheroids was done in the same way as described previously.^66^ Accordingly, the number of FITC labelled cG and cGCM surrounding the large and spheroids was estimated using “Find Spots” building block on Alexa 488 channel. The analysis of the number of spheres in the closest proximity of the small and large spheroids using the various cell: sphere ratios was performed using Harmony 4.8 software (Perkin Elmer). The analysed data obtained was normalized to the highest number of respective sphere interactions at the 1:20 cell: sphere ratio (saturated ratio). Every set of sphere treatment with the spheroids was done in triplicates.

### Microinjection method of the gelatin microspheres and NK cell mimics into zebrafish embryos for the interaction studies

For the injection of gelatin microspheres, the borosilicate glass needles were incubated in 0.01% Poly-L-Lysine (PLL) solution for 30 min. Further, washed twice with Dulbecco’s Phosphate Buffered Saline (DPBS) and used for loading gelatin microspheres. For the injection of NK cell mimics, the borosilicate glass needles were directly used without PLL coating. Further, capillaries were made with the glass needles using Narishige PC-10 dual stage glass micropipette puller (heater level: 65.1). About 1 mg of gelatin microspheres and NK cell mimics were re-suspended in 10 μl of PBS and sonicated for 30 min in water bath sonicator prior to injection. Further, the 5 μl from the mixture was transferred into the capillary using microloader™-microcapillary tips. Finally, the gelatin microspheres or NK cell mimics were injected using a microinjector (PV830 Pneumatic Picopump, with Narishige M-152 manipulator) equipped with a glass capillary.

### *In vivo* interaction studies of gelatin microspheres and NK cell mimics with MDA-MB-231 cells in zebrafish xenograft breast tumour model

Day 1, 2 dpf AB embryos (*N* = 20) were injected with Dil labelled MDA-MB-231 cells at *perivitelline space (PVS)* using borosilicate glass needle and kept for 24 h at 34.9°C using a well-established protocol.^57,67,68^ Briefly, MDA-MB-231 cells were labelled with Vybrant™ DiI lipophilic fluorescent dyes, and re-suspended in RPMI-1640 medium with L-glutamine and sodium bicarbonate at a concentration of 10^6^ cells per 50 μl, and kept on ice before microinjection. Two days post-fertilization (dpf), dechorionated zebrafish embryos were aligned in 2% agarose plate and anesthetized by 2 mg/ml tricaine solution. Approximately 100–200 cells were transplanted into the perivitelline space (PVS) of embryos using a microinjector (PV830 Pneumatic Picopump, with Narishige M-152 manipulator) equipped with a glass capillary. Further, they were checked for cell presence. Fish with fluorescent cells outside the implantation were excluded from further analysis.

After 24 h of MDA-MB-231 cells injection, at day two, dextran-FITC loaded gelatin microspheres (cG) and NK cell mimics (cGCM) were injected on the other side of the PVS site to that of tumour cells (Figure 7(a)). Eventually, the embryos were imaged in Olympus SZX10 fluorescent stereomicroscope equipped with an Olympus DP71 camera at 0 hpi (hour post-injection) of tumour cells, 3 and 24 hpi of gelatin microspheres and NK cell mimics.

There were two possibilities that is, (1) many tumour cells surrounding one gelatin microsphere/NK cell mimic; (2) one tumour cell interacting with more than one gelatin microsphere or NK cell mimics at a time. Therefore, the interaction was analysed in terms of the total number of tumour cells or the total number of spheres in the specific embryos. Two types of analysis have been presented using ImageJ software that is,

(2) $$1.\;\;Area\;of\;tumour\;cells\;interaction\;\left(\%\right)=\frac{Area\;of\;interacted\;tumour\;cells\;\left(yellow\;co-localization\right)}{Area\;of\;total\;tumour\;cells}*100$$

(3) $$\begin{array}{l} 2.\;Area\;of\;gelatin\;microspheres\;or\;NK\;cell\;mimics\;interaction\;\left(\%\right)=\\ \;\;\;\;\;\;\;\;\;\;\;\;\;\;\;\;\;\;\;\;\;\;\;\;\;\frac{Area\;of\;interacted\;gelatin\;microspheres\;or\;NK\;cell\;mimics\left(yellow\;co-localization\right)}{Area\;of\;total\;gelatin\;microspheres\;or\;NK\;cell\;mimics}2.*100 \end{array}$$

### *In vivo* interaction studies of gelatin microspheres and NK cell mimics with macrophages in zebrafish model

At 3 dpf, Kdrl:EGFP Spil:Ds Red zebrafish (*N* = 10) were microinjected with dextran-FITC loaded gelatin microspheres (cG) and NK cell mimics (cGCM) at *perivitelline space (PVS)* of the embryos. At 24 hpi, embryos were monitored in Olympus SZX10 fluorescent stereomicroscope equipped with an Olympus DP71 camera. The embryos were EGFP tagged, and spheres (gelatin microspheres and NK cell mimics) were FITC labelled. Therefore, both blood vessels and spheres were shown in the green channel. The background green fluorescence for the blood vessels was removed for analysis in ImageJ software. This helped in better analysis of the images so as to avoid the overlap of green channels. Further, the yellow co-localized region was quantified, and accordingly, the macrophage uptake or interaction was analysed using ImageJ software and was calculated as below:

(4) $$\begin{array}{l} Macrophage\;interaction\;or\;uptale\;\left(\%\right)=\\ \;\;\;\;\;\;\;\;\;\;\;\;\frac{Area\;of\;interacted\;gelatin\;microspheres\;or\;NK\;cell\;mimics\;\left(yellow\;\;co-localization\right)}{Area\;of\;total\;gelatin\;microspheres\;or\;NK\;cell\;mimics}*100 \end{array}$$

### Loading of sialyltransferase inhibitor (STI) in gelatin microspheres and NK cell mimics

Sialyltransferase Inhibitor, 3Fax-Peracetyl Neu5Ac was used to load into gelatin microspheres (cG) and NK cell mimics (cGCM). First, 0.05 M stock solution of STI was prepared in dimethyl sulphoxide (DMSO) and stored in aliquots at −80°C until use. About 1 mg of gelatin microspheres were suspended and sonicated in 463.5 μl of PBS in glass vial, further 36.5 μl of STI from the stock solution was added, covered the glass vial with aluminium foil and kept on agitation at 100 rpm for 3 h at room temperature. After 3 h, the sample was centrifuged at 14,000 rpm using multifuge X3R centrifuge for 30 min. The supernatant (PBS) was used for estimating the unloaded STI. The pellet (i.e. STI loaded gelatin microspheres) after centrifugation was further used to prepare NK cell mimics.

### Estimation of loading and release of STI using high performance liquid chromatography (HP-LC)

The concentration of 3Fax-Peracetyl Neu5Ac loaded in the spheres was determined using a high performance liquid chromatography (HPLC) system (Shimadzu) equipped with C18 column (250 mm × 4.6 mm) with a constant rate of 1 ml/min and ultraviolent detection at 230 nm. The mobile phase used was 10% acetonitrile to 100% acetonitrile with 0.1% formic acid. The calibration curve for STI was obtained using various concentration from 2 to 0.05 mg/ml in PBS and determined the area under the curve (AUC; Figure S9A). As mentioned in the above section, the supernatant (PBS) was collected and used to evaluate the loaded STI in the gelatin microspheres. The loaded STI was calculated using the equation mentioned below.

(5) AmountofSTIloaded=Initialconc.ofSTI−UnloadedSTIinthesupernatant

The release profile of STI was studied by incubating STI loaded gelatin microspheres and NK cell mimics in 1 ml of PBS at 37°C with 100 rpm agitation. At various time intervals (8, 12, 24, 48, 60 h), 200 μl of PBS was used for HPLC analysis and replaced with 200 μl fresh PBS.

### Treatment of STI loaded gelatin microspheres and NK cell mimics on breast cancer cells (MDA-MB-231) to perform lectin staining

About 20,000 MDA-MB-231 cells/well were seeded in Corning^®^ 96-well flat clear bottom black polystyrene TC-treated microplates for 24 h to adhere the cells properly on the well plate. After 24 h, the old culture medium was removed and washed the cells gently with DPBS. About 1 mg each of STI loaded gelatin microspheres (STI_cG), NK cell mimics (STI_cGCM) and without STI loaded gelatin micropsheres (cG) and NK cell mimics (cGCM) were suspended in 1 ml of culturing media and sonicated for few minutes. About 200 μl of STI_cG, STI_cGCM, cG, cGCM and equivalent amount of STI (200 μM) was incubated with 20,000 MDA-MB-231 cells in each well of 96 well plate for 72 h at 37°C. In parallel, cells without any kind of treatment was also carried out as a control. Every sample incubation was done in triplicates. After 72 h, cells medium was removed and washed twice with 1X Tris-buffered saline (TBS; 10 mM Trizma hydrochloride, 0.1 M NaCl, 1 mM CaCl_2_, 1 mM MgCl_2_, pH 7.2). Cells were fixed with 4% PFA for 15 min and washed four times with TBS. Further, performed lectin staining using an established procedure^69^ with few modifications for staining of cells. Briefly, fixed MDA-MB-231 cells were first blocked with 2% periodic acid-treated BSA in TBS for 1 h at room temperature. Further, the cells were incubated with the 20 μg/ml concentration of fluorescein isothiocyanate (FITC)-labelled lectins in T-TBS (0.05% tween in TBS) in the dark for 3 h at room temperature. FITC-labelled lectin used were: Sambucus nigra (SNA)-I, Peanut agglutinin (PNA), Wheat germ agglutinin (WGA), Maackia amurensis agglutinin II (MAA II), and Wheat germ agglutinin (WGA). After the completion of lectin staining, the cells were imaged Operetta^®^ High Content Imaging System and fluorescence intensity of lectins per cell was determined using in-built harmony 4.8 software installed with operetta instrument.

### Statistical analysis

All statistical analyses used GraphPad Prism 8.00 software. Most data were analysed by One-Way analysis of variance (ANOVA) followed by Tukey multiple comparison test for comparing more than three samples, and two-tailed unpaired *t*-tests for comparing two samples with 95% confidence. *p < 0.05, **p < 0.01, ***p < 0.001, ****p < 0.0001.

## Results

### Characterization of cross-linked gelatin microspheres

Scanning electron microscopic image of the gelatin microspheres after crosslinking (cG) with the 50 mM DMTMM reflects consistent smooth spherical morphology (shown in Figure 1(a)). The parameters used in the synthesis resulted in microspheres with particle size centred on around 10 μm (Figure 1(b)). The free carboxylic group number decreased in the gelatin microspheres after crosslinking, resulted in more positive surface charge from 7.2 ± 0.6 mV(G) to 23.9 ± 1.8 mV(cG) (Figure 1(c)). Using TNBS assay, it was found that the percentage of cross-linking in gelatin microspheres with 50 mM DMTMM was 78.47% ± 0.78%. Calibration curves, derived from diverse concentrations (2–10 mg/ml) of gelatin powder aqueous solution, served to establish accurate correlations for subsequent analyses (Figure S3). With nano-indentation, it was found that increasing DMTMM concentration from 10, 50, and 100 mM resulted in an escalating Young’s modulus, with values of 13.67 ± 0.80 kPa (10 mM DMTMM), 35.05 ± 3.79 kPa (50 mM DMTMM), and 53.13 ± 3.01 kPa (100 mM DMTMM; Figure 1(d)). This tunable elasticity highlights the ability to engineer microspheres with desired mechanical properties. Further, the investigation of microspheres deformation utilizing 50 mM DMTMM within a Lab-on-disc centrifugal microfluidics setup yielded approximately 60.03 ± 9.83% of gelatin microspheres capable of deformation, with an 8 μm gap size chosen for analysis, excluding microspheres with dimensions < 8 μm. The deformable microspheres captured in deformability array and aggregated/smaller size particles < 8 μm non-deformable microspheres captured in v-cups has been presented in Figures 1(f) and (g) and S4. Collectively, these results demonstrate the successful fabrication of mechanically tunable, uniformly spherical, and deformable gelatin microspheres with desirable size, surface, and mechanical characteristics, forming a robust template for subsequent NK cell mimic fabrication.

### Characterization of NK cell mimics

**Figure 2.**
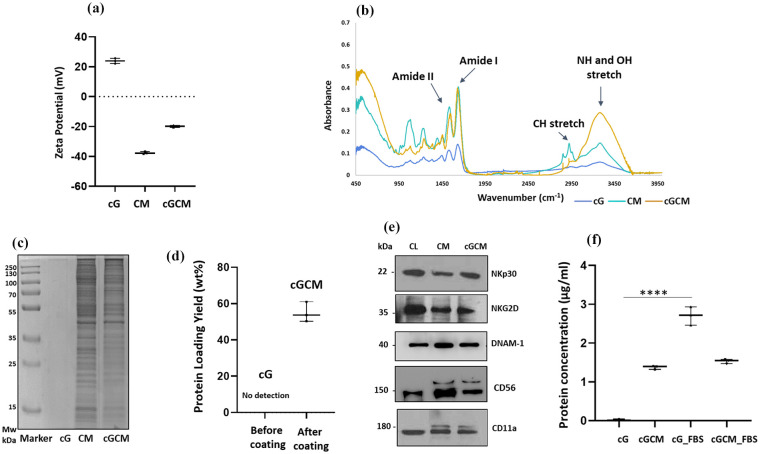
Characterization of NK cell mimics: (a) surface charge on cG, CM and cGCM, *N* = 3, (b) Fourier transform infrared (FT-IR) spectra of cG, CM, and cGCM, (c) SDS-PAGE gel stained with coomassie brilliant blue dye to assess the protein profile on cG, CM and cGCM, (d) Protein loading yield (%) on cGCM, *N* = 3, (e) Western blot to identify specific protein (NKp30, NKG2D, DNAM-1) on CL, CM and cGCM, and (f) Protein absorption analysis on cG and cGCM after incubation in FBS for 3 h at 37°C (cG vs cG_FBS, *p* < 0.0001, 95% CI = −3.004 to −2.355), *N* = 3. Abbreviations: cG: cross-linked gelatin microspheres; cG_FBS: cross-linked gelatin microspheres incubated in foetal bovine serum; cGCM_FBS: NK cell mimics incubated in foetal bovine serum; CL: KHYG-1 cell lysate; CM: isolated KHYG-1 cell membrane; cGCM: NK cell mimics; MW: molecular weight. Graphs are plotted in box and whiskers format with max and min value showing all data points. *p < 0.05. **p < 0.01. ***p < 0.001. ****p < 0.0001.

NK cell mimics were fabricated by coating isolated KHYG-1 cell membrane onto gelatin microspheres using 50 mM DMTMM. The post-assembly zeta potential of gelatin core shifted from +23.9 mV to a negative value (−19.3 mV) due to the KHYG-1 cell membrane (−38.3 mV; Figure 2(a)), confirming successful membrane coating. FT-IR analysis indicated strong peaks in NK cell mimics (cGCM) corresponding to amide I (C=O and N-H) and amide II (N-H and C-N), matching cell membrane intensity. Notably, additional peaks around 2920 and 2850 cm^−1^ appeared due to asymmetric and symmetric stretching of the C-H stretch of the lipid methylene group stretching, and a broad peak (3000–3500 cm^−1^) appeared due to the -N-H stretching motion of peptide backbones of protein amino acids and O-H stretching of carbohydrate polysaccharides in the cell membrane (Figure 2(b)), further confirming the successful transfer of biomolecular components.

**Figure 3.**
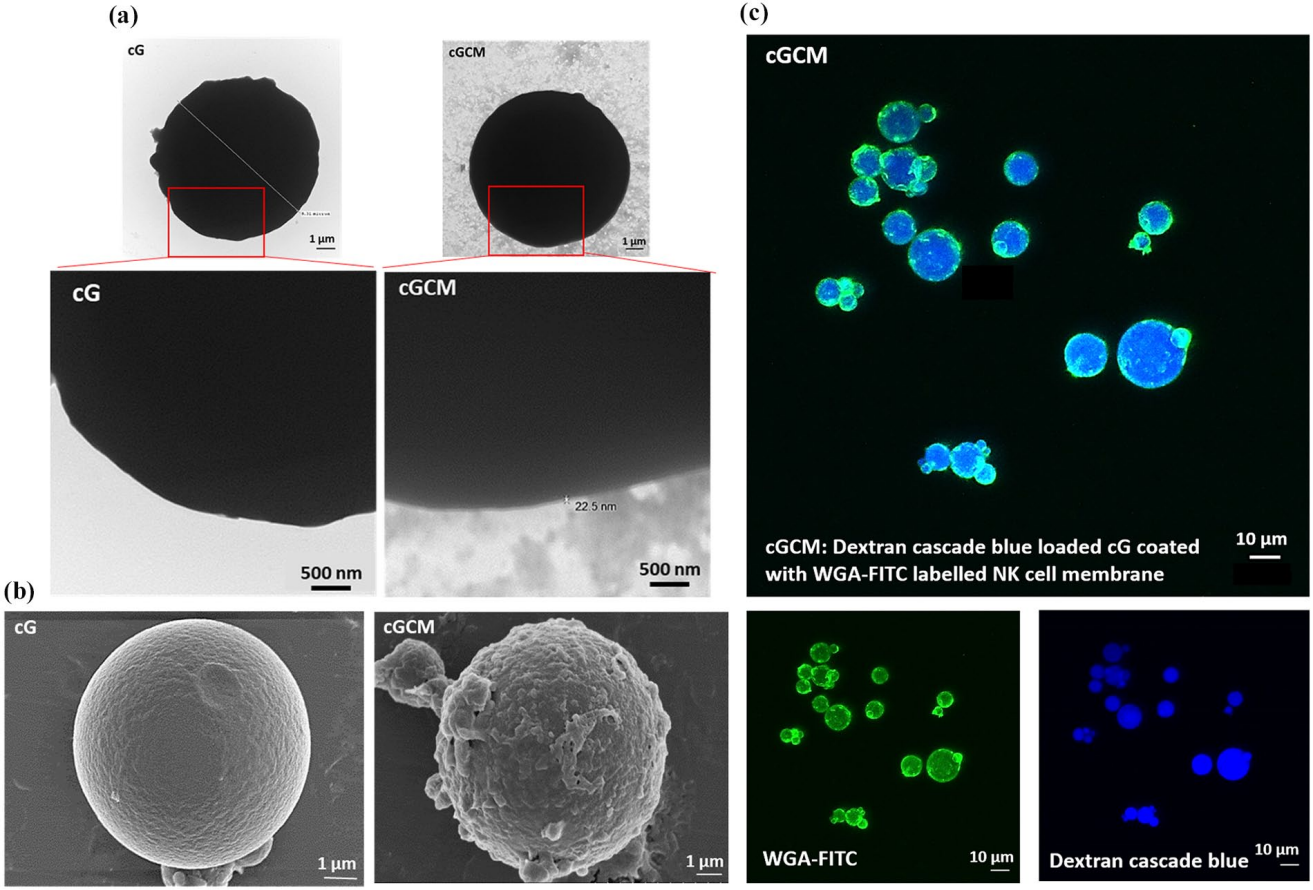
Visualization of NK cell membrane onto gelatin microspheres: (a) transmission electron microscopy (TEM) images of uncoated gelatin microspheres (cG) and NK cell membrane-coated microspheres (cGCM). The cGCM images reveal a dark gelatin core with a surrounding lighter layer corresponding to the NK cell membrane coating. Due to the thin and low-contrast nature of the membrane layer, enlarged views of the red-highlighted regions are included to aid in visualizing coating integrity. Scale bar = 500 nm, (b) field emission scanning electron microscopic (FESEM) images of cG and cGCM, Scale bar = 1 μm, and (c) confocal laser scanning microscopic (CLSM) images of dextran cascade blue loaded gelatin spheres coated with WGA labelled KHYG-1 cell membrane, Scale bar = 10 μm. Abbreviations: cG: cross-linked gelatin microspheres; cGCM: NK cell mimics, WGA-FITC: wheat germ agglutinin-fluorescein isothiocyanate.

SDS-PAGE gel electrophoresis showed gelatin microspheres devoid of protein content before coating. Protein profiles of CM and cGCM closely matched, validating cell membrane protein immobilization on gelatin microspheres (Figure 2(c)). Using equation (1), Protein loading yield analysis revealed around 50 ± 0.5 wt% protein immobilization from CM onto gelatin microspheres (Figure 2(d)), demonstrating efficient membrane transfer. Western blotting verified NK cell protein translocation onto CM and cGCM, including NKp30, DNAM-1, CD11a, NKG2D, and CD56 (Figure 2(e)). FBS incubation analysis indicated a significant amount of protein absorption on cG (2.50 ± 0.05 μg/ml) in comparison to cGCM (0.24 ± 0.02 μg/ml), (cG vs cG_FBS, *p* < 0.0001, 95% CI = −3.004 to −2.355; cGCM vs cGCM_FBS, *p* = 0.4533, 95% CI = −0.4823 to 0.1673) indicating that the cell membrane coating provided effective stealth properties against nonspecific protein binding (Figure 2(f)). Notably, the CM alone was not considered for this study, as it would not provide relevant data for understanding non-specific protein absorption on the surface of cG and cGCM. The focus of this analysis was to assess how the gelatin microspheres and NK cell mimics interacted with the surrounding medium and absorbed proteins, which is critical for evaluating their stability and bio inertness in biological environments. Morphological characterization by TEM revealed a core-shell structure in NK cell mimics, with a dense gelatin core and thin cell membrane coating (Figure 3(a)) with thickness ~22.5 nm. Additional TEM images showing consistent membrane coverage are provided are shown in Figure S5. FESEM images depicted altered surface morphology after cell membrane coating (Figure 3(b)). CLSM further confirmed the complete and uniform membrane coverage on the majority of gelatin microspheres (Figure 3(c)). Collectively, these findings confirm the successful fabrication of NK cell mimics with efficient membrane coating, preserved key membrane proteins, and favourable surface properties that minimize nonspecific protein adsorption. These characteristics are critical for mimicking natural NK cell behaviour and for ensuring immune-evasive performance in subsequent biological evaluations.

### *In vitro* studies with differentiated (diff.) THP-1 cells

**Figure 4.**
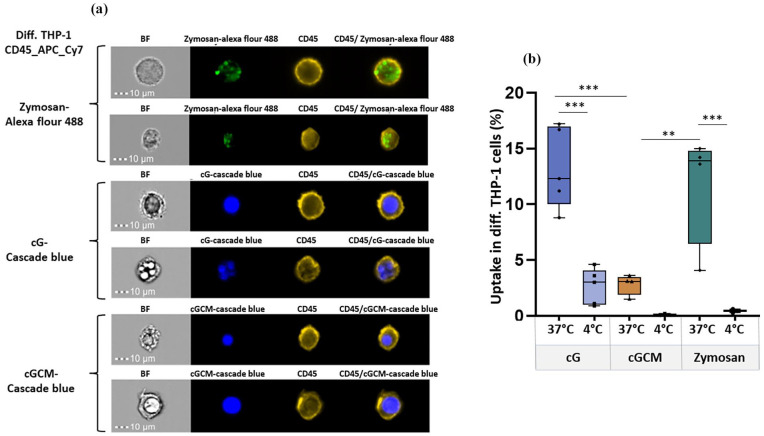
In vitro cellular uptake studies of zymosan A (S. cerevisiae) bioparticles™, gelatin microspheres (cG), and NK cell mimics (cGCM) by differentiated (diff.) THP-1 cells as macrophages for 3 h using Image Stream X (cell/particle: 1:1): (a) For each condition (zymosan, cG–Cascade Blue, and cGCM–Cascade Blue), two representative single-cell uptake images are shown. Each pair includes a brightfield (BF) and the corresponding fluorescence image captured from the same field of view, highlighting the internalization and localization of particles within individual cells at 37°C and (b) comparative analysis of the uptake at 4°C and 37°C (cG vs cGCM, *p* = 0.0004, 95% CI = 4.397 to 16.45; cGCM vs Zymosan (*p* = 0.0035, 95% CI = −15.26 to −2.556), N = 3–5. Zymosan A was used as a positive control and uptake analysis at 4°C was used as a negative control. Zymosan A (S. cerevisiae) bioparticles™ was tagged with Alexa Flour™ 488, cG and cGCM was loaded with dextran cascade blue, diff. THP-1 cells was tagged with CD45_APC_Cy7 dye. Graphs are plotted in box and whiskers format with max and min value showing all data points. *p < 0.05. **p < 0.01. ***p < 0.001. ****p < 0.0001.

**Figure 5.**
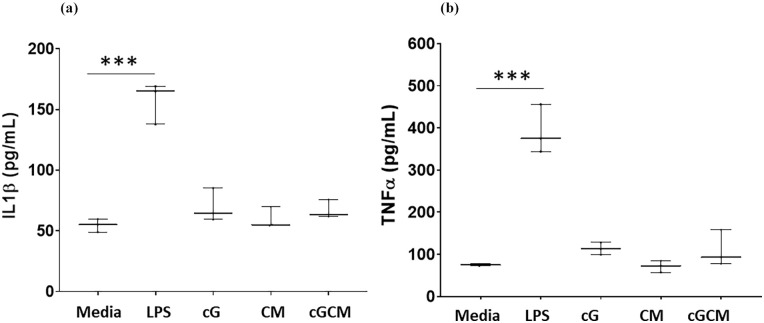
Pro-inflammatory assessment of gelatin microspheres (cG), NK cell mimics (cGCM), NK cell membrane (CM) with differentiated THP-1 cells. Quantitative measurement of pro-inflammatory cytokines, (a) interleukin 1β (IL-1β), *p*< 0.0001, 95% CI [Media vs LPS] = −133.3 to −72.21, (b) tumour necrosis factor α (TNF-α) after 24 h incubation using enzyme-linked immunosorbent assay (ELISA), *p* < 0.0001, 95% CI [Media vs LPS] = −406.8 to −227.1, *N* = 3. Lipopolysaccharides (LPS) was used as positive control. Graphs are plotted in box and whiskers format with max and min value showing all data points. *p < 0.05. **p < 0.01. ***p < 0.001. ****p < 0.0001.

Cytotoxicity assessment of gelatin microspheres (cG) and NK cell mimics (cGCM) against diff. THP-1 cells indicated no toxicity at all concentrations (10–100 µg/ml; Figure S6), demonstrating excellent biocompatbility. In macrophage uptake studies, overlay of intensity based gating strategy showed higher uptake of cG compared to cGCM (Figure S7). Single cell uptake images are shown in Figure 4(a). Based on quantification, cGCM uptake by diff. THP-1 cells was around 10.43% less than cG (Figure 4(b); cG vs cGCM, *p* = 0.0004, 95% CI = 4.397 to 16.45), suggesting that the presence of the NK cell membrane coating reduced recognition and phagocytosis by macrophages. For pro-inflammatory evaluation using ELISA, THP-1 cells treated with cG, CM and cGCM did not induce any significant pro-inflammatory cytokine (IL-1β and TNF-α; Figure 5(a) and (b)), indicating that these systems do not activate inflammatory pathways. Overall, these results collectively indicate that NK cell mimics (cGCM) exhibit excellent biocompatibility, reduced phagocytic uptake, and no pro-inflammatory activation, supporting their potential as immune-evasive platforms for therapeutic applications.

### *In vitro* interaction studies with 2D cultures of breast cancer cells (MDA-MB-231)

**Figure 6.**
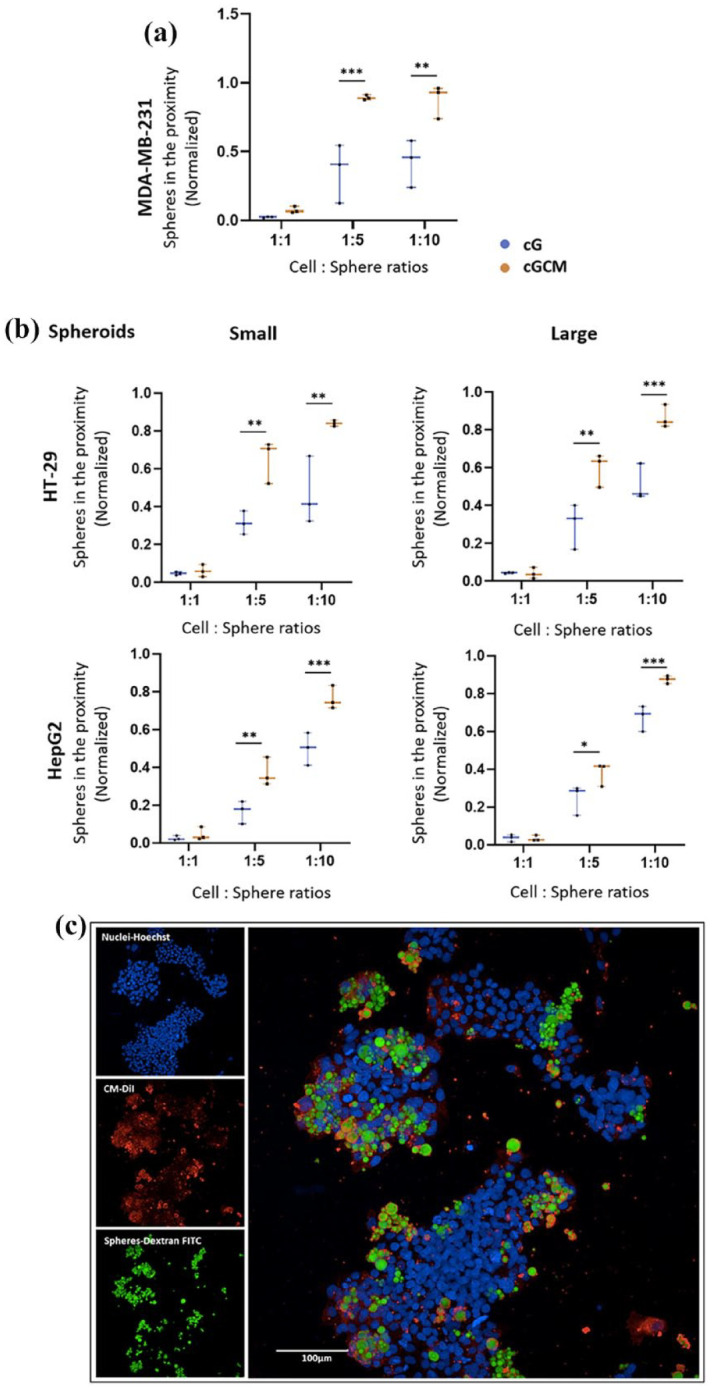
In vitro interaction studies of gelatin microspheres (cG) and NK cell mimics (cGCM) with 2D cultures of MDA-MB-231 (human breast cancer cell line) and 3D spheroids of HT-29 (human colon cancer cell line) and HepG2 (human liver cancell cell line) using various cell: sphere ratios (1:1, 1:5, 1:10, 1:20) after 24 h incubation: (a) comparative analysis of number of cG and cGCM interacting or in the close proximity with the MDA-MB-231(One-way ANOVA, cG vs cGCM (1:5), *p* = 0.0003, 95% CI = −0.8344 to −0.2330; cG vs cGCM (1:10), *p* = 0.0018, 95% of CI: −0.7513 to −0.1499), *N* = 3, (b) comparative analysis of number of cG and cGCM in close proximity to spheroids of HT-29 small spheroid (one-way ANOVA, cG vs cGCM (1:5), *p* = 0.0019, 95% CI = −0.5650 to −0.115; cG vs cGCM (1:10), *p* = 0.0014, 95% CI: −0.5752 to −0.1216); HT- 29 large spheroid (one-way ANOVA, cG vs cGCM (1:5), *p* = 0.0011, 95% CI = −0.4875 to −0.1083; cG vs cGCM (1:10), *p* = 0.0002, 95% CI: −0.5436 to −0.1644), and HepG2 small spheroid (one-way ANOVA, cG vs cGCM (1:5), *p* = 0.0040, 95% CI = −0.3505 to −0.05568; cG vs cGCM (1:10), *p* = 0.0010, 95% CI: −0.4110 to −0.1162); HepG2 large spheroid (one-way ANOVA, cG vs cGCM (1:5), *p* = 0.0325, 95% CI = −0.2586 to −0.008217; cG vs cGCM (1:10), *p* = 0.0010, 95% CI: −0.3244 to −0.07408), *N* = 3; *p < 0.05, **p < 0.01, ***p < 0.001, ****p < 0.0001, and (c) representative 2D image of interaction of cGCM with HT-29, 1:5 (cell: sphere ratio), NK cell membrane was tagged with CM-DiI dye, NK cell mimics were loaded with dextran-FITC, and nuclei of HT-29 with Hoechst, Scale bar = 100 μm. Graphs are plotted in box and whiskers format with max and min value showing all data points.

Automated quantitative high-content analysis (Figure 6(a)) revealed enhanced interaction/ proximity of cGCM with MDA-MB-231 cells compared to cG alone with increase in cell-to-spheres ratios. For 1:5 ratio (*p* = 0.0003, 95% CI [cG vs cGCM] = −0.8344 to −0.2330), cGCM displayed ~53.37% more interaction/ proximity with breast cancer cells compared to cG alone. For 1:10 (*p* = 0.0018, 95% of CI [cG vs cGCM]: −0.7513 to −0.1499), cGCM displayed ~45.05% more interaction/ proximity with breast cancer cells compared to cG alone. The representative image of interaction of cGCM with MDA-MB-231 cells is shown in Figure S8. These findings demonstrate that NK cell membrane coating significantly improves the targeting and adherence capabilities of the gelatin microspheres towards cancer cells, supporting the hypothesis that biomimetic surface features facilitate closer and more efficient interactions with tumour cells. This enhanced interaction is crucial for potential downstream applications such as targeted delivery or modulation of the tumour microenvironment.

### *In vitro* interaction studies with 3D spheroids of liver (HepG2) and colon (HT-29) cancer cells

Automated quantitative high-content analysis (Figure 6(b)) revealed enhanced interaction/proximity of cGCM with HepG2 and HT-29 spheroids compared to cG alone, at various cell-to-sphere ratios. For HepG2 (1:5 ratio), cGCM demonstrated 14.38% and 29.35% more close proximity to small (*p* = 0.0040; 95% CI [cG vs cGCM]: −0.3505 to −0.0556) and large spheroids (*p* = 0.0325; 95% CI [cG vs cGCM]: −0.2586 to −0.008217), respectively, compared to cG alone. For HepG2 (1:10 ratio), cGCM demonstrated 22.13% and 18.19% more close proximity to small (*p* = 0.0010; 95% CI [cG vs cGCM]: −0.4110 to −0.1162) and large (*p* = 0.0010; 95% CI [cG vs cGCM]: −0.3244 to −0.07408) spheroids, respectively, compared to cG alone. For HT-29 (1:5 ratio), cGCM demonstrated 32.65% and 24.14% more close proximity to small (*p* = 0.0019; 95% CI [cG vs cGCM]: −0.5650 to −0.115) and large (*p* = 0.0011; 95% CI [cG vs cGCM]: −0.4875 to −0.1083) spheroids, respectively, compared to cG alone. For HT-29 (1:10 ratio), cGCM demonstrated 26.64% and 24.51% more close proximity to small (*p* = 0.0014; 95% CI [cG vs cGCM]: −0.5752 to −0.1216) and large (*p* = 0.0002; 95% CI [cG vs cGCM]: −0.5436 to −0.1644) spheroids, respectively, compared to cG alone. Visual inspection of the images used to generate these data also supported the notion of greater proximity of cGCM’s to the spheroids models, compared cGs alone. Analysis of images also revealed NK cell membrane retention on the spheres, likely pointing to its enhance stability and interaction towards cancer cells. (Figure 6(c)). These results reinforce that NK cell membrane coating facilitates improved interaction of microspheres with 3D tumour models, a critical feature for their potential application in targeted cancer therapy. The preserved membrane coating further emphasizes the system’s robustness in dynamic biological environments.

### Interaction studies of gelatin microspheres (cG) and NK cell mimics (cGCM) with MDA-MB-231 cells in zebrafish xenograft breast tumour model

**Figure 7.**
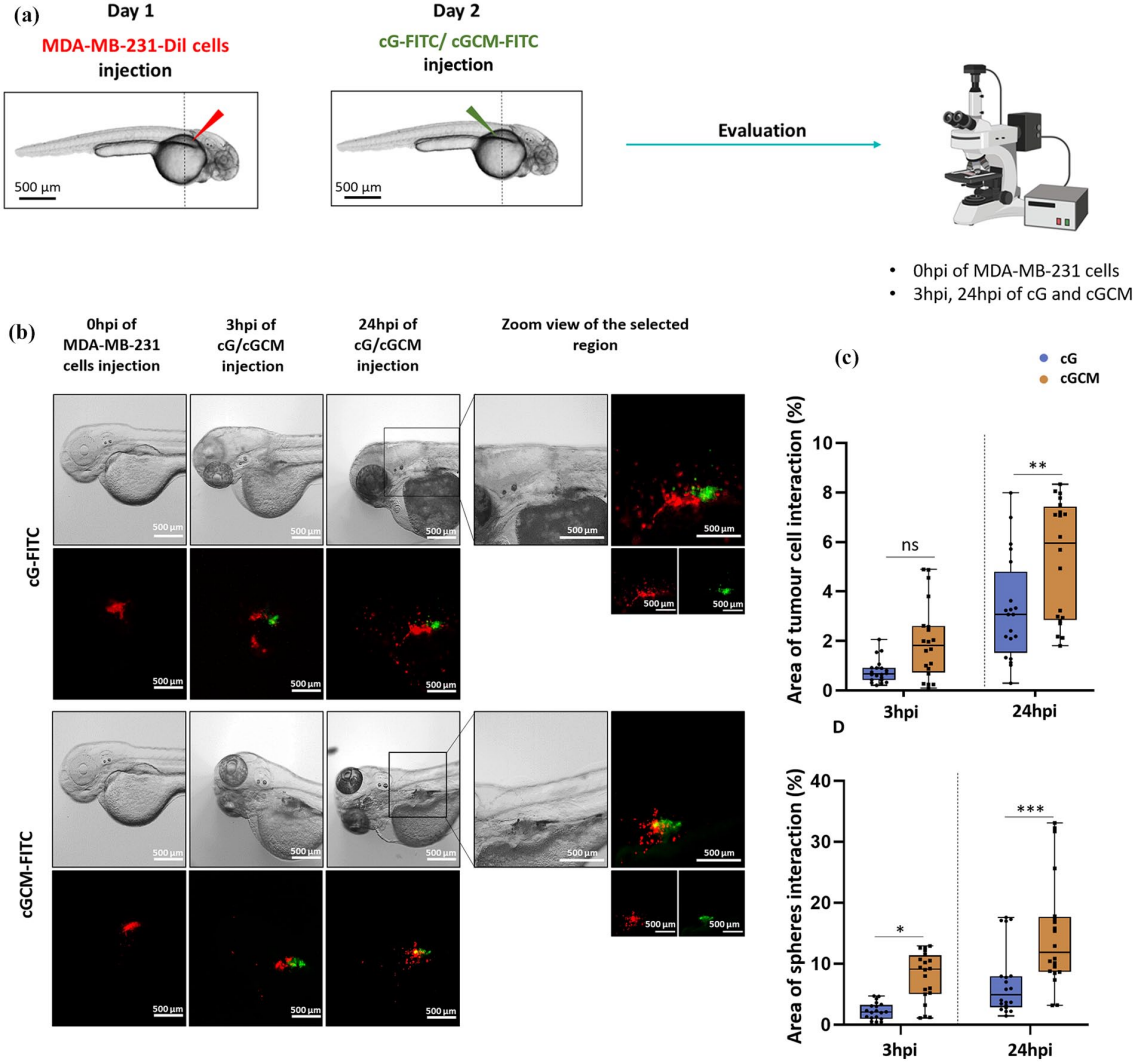
In vivo interaction studies with MDA-MB-231 (breast cancer cell line) in zebrafish xenograft breast tumour model: (a) schematics of the site and time of injection of MDA-MB-231 cells, dextran-FITC loaded gelatin microspheres and NK cell mimics, (b) microscopic images (bright field, red and green fluorescence) of same embryos at 0 h post injection (hpi) of MDA-MB-231 cells, at 3 and 24 hpi of dextran-FITC loaded gelatin microspheres (cG) and NK cell mimics (cGCM), Scale bar = 500 μm, Zoom view of the selected region is also presented for 24 hpi, Scale bar = 500 μm, (c) quantification of the interaction with respect to tumour cells (One-way ANOVA, cG vs cGCM (24 hpi), *p* = 0.0032, 95.00% CI = −3.496 to −0.5426), and (d) with respect to spheres (One way ANOVA, cG vs cGCM (3 hpi), *p* = 0.0131, 95% CI = −10.51 to −0.9147, cG vs cGCM (24 hpi), *p* = 0.0002, 95% CI = −12.73 to −3.131), *N* = 20. *Note.* No cytotoxic payload was incorporated in these experiments, as the primary aim was to evaluate the interaction of NK cell mimics with tumour cells—specifically their attachment and spatial proximity—rather than inducing tumour cell death or inhibiting spheroid growth. Graphs are plotted in box and whiskers format with max and min value showing all data points. *p < 0.05. **p < 0.01. ***p < 0.001. ****p < 0.0001.

Quantification analysis reveals that at 24 hpi, a more significant interaction of tumour cells with cGCM was observed, as shown in Figure 7(b). Using equation (2), based on the quantification with respect to tumour cells (Figure 7(c)), at 24 hpi (one-way ANOVA, *p* = 0.0032; 95% CI [cG-cGCM]: −3.496 to −0.5426), around 5.91% ± 2.34% area of tumour cells interacted with NK cell mimics and 2.37 ± 1.67% area of tumour cells interacted with gelatin microspheres. Using equation (3), based on the quantification with respect to spheres (Figure 7(d)), at 24 hpi (One-way ANOVA, *p* = 0.0002; 95% CI [cG-cGCM]: −12.73 to −3.131), around 14.96% ± 9.15% area of NK cell mimics and 7.03% ± 5.62% area of gelatin microspheres interacted with tumour cells. Hence, a significant interaction of NK cell mimics with tumour cells were observed, which was significantly higher than gelatin microspheres alone at 24 hpi. These results clearly indicate that NK cell membrane coating significantly enhanced the interaction of the microspheres with tumour cells in vivo within the zebrafish breast cancer xenograft model. The increased tumour cell interaction highlights the functional advantage conferred by the NK cell membrane coating, supporting the concept that biomimetic engineering improves tumour-targeting capabilities even under dynamic and complex in vivo conditions. This is a crucial step towards developing effective cell mimic-based therapeutic strategies.

### Interaction studies of gelatin microspheres and NK cell mimics with macrophages in zebrafish

**Figure 8.**
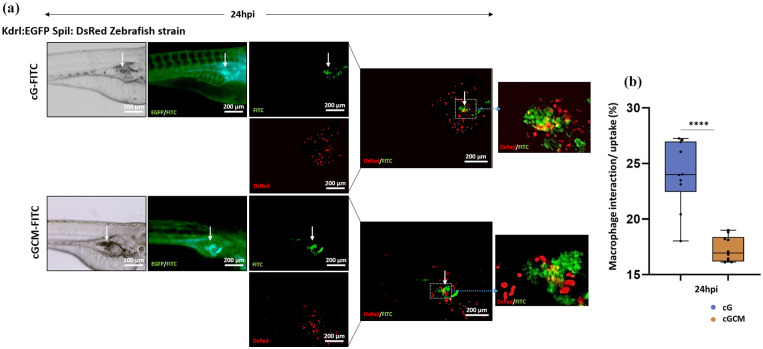
In vivo interaction/cellular uptake studies of gelatin microspheres (cG) and NK cell mimics (cGCM) with macrophages in zebrafish model: (a) microscopic images (bright field, red and green fluorescence) presenting the interaction at 24 h post injection (hpi) of dextran-FITC loaded gelatin microspheres (cG) and NK cell mimics (cGCM) with DsRed macrophages in 3 day post fertilized (dpf) Kdrl:EGFP Spil:DsRed Zebrafish strain model, Scale bar = 200 μm. The zebrafish embryos’ blood vessel were EGFP tagged, and spheres were FITC labelled. Therefore, both blood vessels and spheres were shown in the green channel. The background green fluorescence for the blood vessels was removed in Image J for analysis and marked the position of the spheres with white arrow for better analysis of the images to avoid the overlap of green channels, and (b) quantification of interaction/cellular uptake of cG and cGCM with macrophages at 24 hpi of spheres (unpaired *t*-test, cGCM vs cG, *p < 0.0001, 95.00% CI = −8.894 to −4.596), *N* = 10. Graphs are plotted in box and whiskers format with max and min value showing all data points. *p < 0.05. **p < 0.01. ***p < 0.001. ****p < 0.0001.

Quantification analysis reveals that at 24 h post injection (hpi), the macrophages were found closer to or crowded near the gelatin microspheres more than compared to the NK cell mimics, as shown in Figure 8(a). This facilitates more interaction or uptake of the gelatin microspheres compared to NK cell mimics. Based on the quantification (Figure 8(b)), using equation (4) it was found that at 24 hpi cG (24.04% ± 3.02%) interacted with or were taken up by macrophages, which was significantly more than what was seen for cGCM (17.50% ± 1.10%). The significantly reduced interaction of cGCM microspheres with macrophages compared to uncoated cG microspheres (*p* < 0.0001; 95% CI [cGCM-cG]: −8.894 to −4.596) suggests the ability of NK cell mimics to evade macrophage recognition and uptake more effectively than uncoated gelatin microspheres, supporting the design rationale of surface engineering to prolong systemic circulation and reduce premature clearance—key attributes for enhancing tumour targeting efficiency in vivo.

### Sialyltransferase inhibitor (STI, 3Fax-peracetyl Neu5Ac) studies

**Figure 9.**
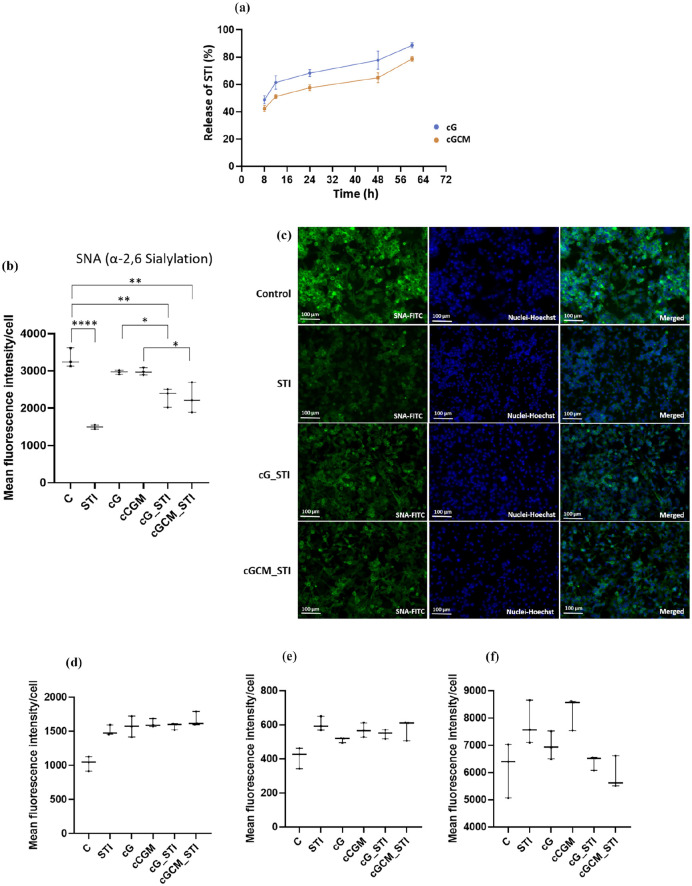
Sialyltransferase inhibitor (STI, 3Fax-Peracetyl Neu5Ac) studies: (a) Release of STIanalysis from gelatin microspheres (cG) and NK cell mimics (cGCM) using a high-performance liquid chromatography (HPLC) system, *N* = 3, Effect of STI, STI loaded gelatin microspheres (cG_STI), and STI loaded NK cell mimics (cGCM_STI) on MDA-MB-231 cells. Mean fluorescence intensity/cell of (b) Sambucus nigra (SNA) lectin (C vs STI, *p*< 0.0001, 95% CI = 2458–1208; C vs cG_STI, *p* = 0.0015, 95% CI = 644 to 393.5; C vs cGCM_STI, *p* = 0.0011, 95% CI = 1686 to 436.2; cG vs cG_STI, *p* = 0.0363, 95% CI = 1286–35.53; cGCM vs cGCM_STI, *p* = 0.0228, 95% CI = 1337 to 86.87), (c) fluorescence microscopic images of sambucus nigra (SNA) lectin expression, (d) peanut agglutinin (PNA) lectin, (e) Maackia amurensis (MAA) lectin, and (f) qheat germ agglutinin (WGA) lectin expression after treatment of 200 μM STI, cG, cGCM, cG_STI, cGCM_STI for 72 h at 37°C, *N* = 3. Abbreviations: C: Control (cells with only media), cG: gelatin microspheres, cGCM: NK cell mimics. *Note.* No cytotoxic payload was incorporated in these experiments. Note: This study aimed to assess the controlled release of functional glycan modulators, not tumour cell killing or spheroid growth inhibition. Graphs are plotted in box and whiskers format with max and min value showing all data points. *p < 0.05. **p < 0.01. ***p < 0.001. ****p < 0.0001.

The optimization of STI detection via HPLC yielded a characteristic peak ~16.29 min (Figure S9B), with a limit of detection (LOD) of 50 μg/ml. HPLC analysis demonstrated that ~130 ± 6.2 μg STI could be loaded per 1 mg of cG (using equation (5)). Detectable STI release from cG was observed after 8 h, with a higher release percentage compared to cGCM (Figure 9(a)). After 12 h, cG released around 61.37 ± 4.00% STI, while cGCM released approximately 51.13% ± 1.18%. The release profile of STI from both cG and cGCM exhibited a progressive increase over time, although cG consistently released slightly more STI than cGCM at all measured intervals. Based on quantification of lectin fluorescence intensity on MDA-MB-231 cells (Figure 9(b)), treatment with free STI, STI-loaded gelatin microspheres (cG_STI), and STI-loaded NK cell mimics (cGCM_STI) resulted in a significant decrease in α-2,6-sialylation in MDA-MB-231 cells, as measured by reduced SNA lectin binding. Compared to untreated controls, STI (*p* < 0.0001, 95% CI = 2458–1208), cG_STI (*p* = 0.0015, 95% CI = 644–393.5), and cGCM_STI (*p* = 0.0011, 95% CI = 1686–436.2) all significantly lowered fluorescence intensity. Encapsulation of STI within microspheres also reduced sialylation compared to blank spheres (cG vs cG_STI, *p* = 0.0363; cGCM vs cGCM_STI, *p* = 0.0228), confirming functional release and activity of the loaded inhibitor. Microscopic images confirmed decreased FITC fluorescence channel intensity for SNA in STI, cG_STI, and cGCM_STI-treated cells compared to untreated cells (Figure 9(c)). Notably, there was no observed effect on the fluorescence intensity of PNA, WGA, and MAA lectins after STI/cG_STI/GCM_STI treatment (Figure 9(d)–(f)), suggesting specific inhibition of α-2,6 sialylation without off-target glycosylation effects. These results validate that STI-loaded NK cell mimics successfully deliver functionally active sialylation inhibitors to target cells, achieving selective modulation of cell-surface glycosylation—a critical step towards potential therapeutic applications in cancer treatment.

## Discussion

The overarching goal of this study is to integrate the surface characteristics, size, and relevant cell-like mechanical properties into the designed NK cell mimics. The mimics are assembled using cell membrane from KHYG-1 (human NK cell line) cells onto cross-linked gelatin microspheres as template. The selection of KHYG-1 cell line offered two main advantages: first, their close resemblance to primary NK cells and presence of key activating receptors (DNAM-1, NKp30, NKG2D), adhesion receptor (CD11a) and NK cell identifying receptor (CD56) on their surface that help target and adhesion to tumour cells.^70–72^ Second, these cells were readily expandable in vitro,^29–31^ ensuring a scalable source of membrane for crafting NK cell mimics. The choice of gelatin was also dictated by two factors: first, Gelatin A, high bloom (300) was used for microsphere fabrication to facilitate stronger gelation. Second, isoionic range of Gelatin A (porcine) 6.5–9 is within the ideal pH range for amide bond formation and facilitates modulation of mechanical properties via DMTMM cross-linking to design NK cell mimics.^73,74^ For microsphere cross-linking we deviated from the traditional counterparts like glutaraldehyde^59,75^ owing to its toxicity risk and instability and reactivity of the resulting Schiff base intermediate.^76,77^ DMTMM is a non-toxic, water soluble, zero-length cross-linker, widely used in both chemical and biochemical scenarios.^32,33^ It combines active ester and amide bond formation in a single step (Figure S10) and can be used over a wider pH range producing water soluble by-products.^78^ This cross-linking system is a significant improvement over other systems in terms of its cytocompatibility this making it a potential alternative to widely used EDC/NHS chemistry.^79^

Microspheres showed an increase in positive surface charge post cross-linking suggesting a decrease in free carboxylic groups while maintaining a consistent spherical morphology (Figure 1(a) and (c)). Their particle size remained centred around 10 μm (Figure 1(b)), comparable to NK cells’ size.^80^ While such microscale dimensions may be considered large for systemic delivery, this design is intentional and aligns with our goal of mimicking the size and behaviour of natural immune effector cells. This scale enables biologically relevant cell–cell interactions, tumour retention, and engagement with the tumour microenvironment. Moreover, our platform is intended for localized administration—via intratumoral, peritumoral, or other regional routes—which circumvents the limitations of systemic delivery. Such approaches are well-established in clinical oncology and compatible with larger particles. Indeed, several clinically approved microsphere-based systems (e.g. for embolization or depot-based drug delivery) operate successfully within this size range. Thus, although systemic administration poses challenges for microparticles, our platform is specifically designed for local delivery, where the chosen size supports both functional mimicry and translational feasibility.

While the bio-distribution and circulation times of RBCs are well known to benefit from their elastic properties,^81,82^ the aim of our study was not to directly replicate RBC function but rather to use RBCs conceptually to highlight the mechanical advantage provided by elasticity. RBCs are highly deformable, allowing them to traverse narrow capillaries and evade phagocytic clearance. Our objective was to tune the elastic modulus of the gelatin microspheres to replicate these favourable mechanical properties, which are commonly associated with RBCs and other deformable particles. Elastic modulus (mechanical properties) of gelatin is dependent on three factors: bloom strength, concentration and/or degree of cross-linking and temperature. To achieve elastic properties (Young’s modulus) closer to RBCs at physiological temperature (37°C), we selected a high bloom porcine gelatin and varied DMTMM concentration (10, 50, and 100 mM) for cross-linking. Our approach demonstrates the potential for gelatin microspheres to possess a tunable elasticity, enabling us to modulate mechanical properties and optimize their functionality for use in drug delivery systems. Young’s modulus of microspheres using 50 mM DMTMM concentration (35.05 ± 3.79 kPa) was closest to the values reported for RBCs (26 ± 7 kPa).^83^ While the selected Young’s modulus (~35 kPa) of cG microspheres exceeds the typical range reported for resting immune cells—which generally exhibit moduli in the kPa range, often below 10 kPa depending on cell type and measurement technique—it is important to recognize that immune cell stiffness is highly dynamic and context-dependent. Upon activation, immune cells can undergo cytoskeletal remodelling that increases their mechanical stiffness several-fold, with some studies reporting moduli approaching or exceeding 20 kPa depending on the activation state and assay conditions.^84–86^ This mechanoadaptive behaviour allows immune cells to resist mechanical stress during transmigration, synapse formation, or cytolytic engagement. Our aim was not to mimic a specific immune cell modulus in a single state, but to capture functionally relevant mechanical characteristics that support circulation, tissue infiltration, and resilience under physiological forces. Therefore, we tuned the stiffness of our gelatin microspheres using high-bloom gelatin and optimized cross-linking to closely match the elasticity of red blood cells (~26 kPa) rather than quiescent immune cells. RBCs were chosen as a mechanical benchmark due to their well-characterized deformability, long circulation half-life, and immune evasiveness. The resulting cG microspheres (~35 kPa) represent a strategic compromise—rigid enough to resist shear degradation and maintain integrity during circulation, yet soft enough to traverse capillary-sized constrictions, as validated in our microfluidic deformability assays, where ~60.03% ± 9.83% of cG microspheres were able to pass through 8 μm constrictions, supporting their capacity to navigate narrow vascular structures. This alignment of the young’s modulus of cG (50 mM DMTMM) with RBCs promoted their selection as a template for assembling NK cell mimics. In the study, we have measured Young’s modulus at physiological temperature, which provides a robust indication of the elasticity of the NK cell mimics. This parameter is closely related to key biological processes such as circulation time, tumour penetration, and immune system evasion. We believe that the elasticity of the system plays a significant role in influencing these functions, as stiffer particles are generally cleared more rapidly by the mononuclear phagocyte system (MPS), while softer particles have the potential to evade immune detection for longer periods and penetrate tumours more efficiently. While the current study primarily focuses on the elasticity of the NK cell mimics, as measured by Young’s modulus, we recognize that incorporating viscoelastic properties could provide further insights, particularly in dynamic environments. Viscoelasticity reflects both the elastic (reversible) and viscous (time-dependent) responses of the system, which could influence cellular interactions, immune evasion, and tumour penetration. The inclusion of viscoelastic parameters could be particularly beneficial for understanding the time-dependent behaviour of the mimics during circulation or when subjected to shear stress in the bloodstream. Future studies exploring viscoelasticity could offer a more comprehensive characterization of the NK cell mimics and provide a deeper understanding of how mechanical properties influence their therapeutic potential.

The final step in the assembly process involved coating cell membrane onto the gelatin microspheres. Homogenous dispersions of cG and isolated KHYG-1 cell membrane in water were individually prepared by sonication, stabilized due to the surface positive and negative charges of cG and cell membrane respectively. The dispersions were mixed and sonicated to assemble the NK cell mimics (cGCM). Electrostatic interactions between cG and cell membrane (CM) can be attributed to the stability of this final assembly. Noticeable shift in the zeta potential of the gelatin core from positive to negative confirmed coating of cell membrane (Figure 2(a)). Furthermore, we employed FT-IR, for qualitatively comparison of cG, CM and cGCM. Intensity of amide I, II, C-H, N-H, and O-H stretching was more prominent in the IR spectra for cGCM confirming the presence of cell membrane components, such as phospholipids, proteins, and carbohydrates post assembly (Figure 2(b)). Protein profiles of cG, CM, and cGCM post assembly were compared using SDS-PAGE gel electrophoresis. RIPA, a standard buffer used for protein extraction cannot lyse proteins in gelatin,^87–89^ indicated by the absence of bands for cG in SDS-PAGE, confirmation that bands in cGCM were solely from the coated cell membrane. The protein profiles of CM and cGCM closely matched, unequivocally confirming the successful immobilization of cell membrane proteins onto the gelatin microspheres (Figure 2(c)). Notably, the intensity of protein bands in cGCM was lower than that of CM. Quantification of protein loading revealed that approximately 50% of the proteins from CM were successfully immobilized onto the gelatin microspheres (Figure 2(d)). Western blotting analysis demonstrated the preservation and translocation of essential receptors (NKp30, NKG2D, DNAM-1, CD11a, CD56) from isolated cell membranes to NK cell mimics (Figure 2(e)). Cells are known to elicit biological functions by exclusive interactions with its physiological environment via its surface receptors, in contrary gelatin is prone to non-specific interactions.^90,91^ An effective way of confirming the presence of cell membrane on cGCM is to evaluate this behaviour in presence of a generic protein like FBS. Comparing the final protein content of cG and cGCM post FBS incubation revealed reduced non-specific binding for the mimics demonstrating their specificity and shielding of gelatin. (Figure 2(f)). Visual assessment of coating was carried out using microscopic techniques such as FESEM, TEM and CLSM (Figure 3). FESEM images transformed surface of cG from smooth to coarse following cell membrane coating, providing an initial readout into the coating process. Examination of transmission electron microscopy (TEM) images of cG and cGCM (Figures 3(a) and S5) display a dense, dark gelatin core surrounded by a lighter NK cell membrane coating in the nanometre range, consistent with literature reports for a core shell morphology. Given the microscale size and contrast of the gelatin core, identifying the thin membrane layer can be challenging. To aid visualization, enlarged views of the red-highlighted regions are provided to better illustrate the coating integrity. Lastly, CLSM offered insights into the overall efficiency of the coating process, where cG and CM were labelled with two different dyes that is, dextran cascade blue and WGA-FITC, respectively. The fluorescent colocalization demonstrated that the majority of gelatin microspheres were effectively coated with the cell membrane. It is important to note that the NK cell mimic membrane coating, with a thickness of nanometre, does not influence the elastic properties of the gelatin microspheres. Given the thin nature of the membrane, its contribution to the mechanical properties of the microspheres is negligible. The elasticity of the microspheres is primarily determined by the gelatin matrix, which is responsible for their bulk mechanical behaviour, including deformability and elasticity. The cell membrane coating plays a critical role in imparting biological functionality, such as receptor presentation for tumour targeting, without significantly altering the overall mechanical properties of the microspheres.

Introduction of polymer-based delivery systems into biological circulation triggers a foreign body reaction and clearance driven by macrophages via phagocytosis. Their rate of clearance depends on surface properties and size.^92^ Cell membrane coating was carried out to incorporate stealth properties into our delivery system by evading foreign body reaction thereby increasing circulation times. To validate integration of this property into our NK cell mimics we compared the uptake of cG and cGCM in presence of differentiated THP-1 cells. THP-1 are well established cell lines to study monocyte/macrophage differentiation and function.^93^ The visualization and quantification was carried out using Image StreamX. A technology that combines the features of both flow cytometry and fluorescence microscopy.^94^ CD45, a receptor exclusive to differentiated THP-1 was selected to tag the macrophages.^95^ ImageStream X, with its visual data capabilities, addresses this issue by enabling the capture of high-quality images showing the uptake of microspheres by individual cells, improving gating accuracy, population mapping and aiding in the validation of data analysis results, and reducing the risk of misinterpretation.^96,97^ It is noteworthy that at 4°C, where cellular processes such as receptor-mediated endocytosis are significantly reduced, gelatin microspheres exhibited some level of uptake, likely due to passive adsorption rather than active uptake mechanisms. This was in contrast to Zymosan and NK cell mimics, which showed almost no uptake under these conditions. The lack of Zymosan and NK cell mimics uptake at 4°C highlights the differences in the surface properties of these particles. Despite the limited uptake at 4°C, at 37°C—where active endocytosis occurs—gelatin microspheres exhibited a higher level of uptake than NK cell mimics. Our studies showed that macrophage uptake of cG was 10.43% higher than cGCM (Figure 4). This confirmed the integration of macrophage eluding stealth properties into the NK cell mimics post assembly.

Additionally, NK cell mimics were evaluated for pro-inflammatory response and biocompatibility. cG and cGCM were co-incubated with macrophages (diff. THP-1 cells) and the secretion of pro-inflammatory cytokines, such as TNF-alpha and IL-1beta, quantified by ELISA. Our assessment revealed absence of pro-inflammatory cytokines in the cultures and good biocompatibility for both cG and cGCM (Figures 5 and S6). Overall, these results demonstrate that the NK cell mimics (cGCM) are biocompatible, exhibit reduced immune recognition, and do not activate inflammatory pathways. These properties are highly desirable for their potential use in cancer immunotherapies, where immune evasion is a critical factor in prolonging therapeutic efficacy and reducing side effects. Additionally, their ability to maintain non-inflammatory characteristics while interacting with cancer cells positions these mimics as a promising platform for drug delivery, particularly for targeted therapies that require long circulation times and minimal interference from the immune system. To explore the basis for this reduced uptake, we considered known immunological mechanisms. NK cell membranes present “self” markers, such as MHC class I molecules, which can be recognized by monocytes/macrophages and interpreted as autologous, reducing the likelihood of phagocytic engagement. In addition, NK cell membranes lack immunogenic or pathogen-associated molecular patterns (PAMPs) and danger-associated molecular patterns (DAMPs), thereby avoiding activation of pattern recognition receptors (PRRs) on macrophages. The native lipid and glycoprotein composition of NK cell membranes may also reduce non-specific protein adsorption (observed in our protein absorption studies) and opsonization, key steps in macrophage recognition. Importantly, our cytokine analysis confirmed that the cGCMs did not trigger pro-inflammatory signalling, indicating a non-activating or immunologically silent profile. This supports the concept that the biomimetic coating not only physically shields the core but also functionally attenuates macrophage activation. While we acknowledge that we did not perform receptor-ligand inhibition assays in the current study, such as blocking MHC-I or scavenger receptors, we recognize that these would provide additional mechanistic clarity. Future studies are planned to dissect the contributions of specific receptor-ligand interactions, particularly at the immunological synapse between THP-1 cells and cGCMs. Nevertheless, the observed reduction in uptake, combined with the absence of inflammatory response, provides strong evidence that NK cell membranes endow stealth characteristics through a combination of self-marker presentation, reduced opsonization, and immune-inert surface mimicry.

As part of tumour annihilating mechanism, NK cells recognize and target tumorous cells using their surface receptors; DNAM-1, NKp30, NKG2D.^98,99^ During the assembly it is vital that these surface receptors are conserved and transferred into the final mimics to elicit NK cell like biological function. We verified the presence of these receptors on cGCM using western blotting analysis. Subsequently, their ability to interact with tumour cells in a conventional 2D cancer cell culture. We examined these interactions using MDA-MB-231 breast cancer cells at varying cell-to-spheres ratios (1:1, 1:5, and 1:10). Notably, cGCM exhibited higher proximal density to MDA-MB-231 cells when compared to cG (Figure 6(a)). This enhanced interaction can be attributed to the expression of tumour-associated ligands on MDA-MB-231 cells, including CD155 and Nectin-2 (ligands for DNAM-1)^100,101^, B7-H6 (NKp30 ligand),^102^ and ULBP-4 (NKG2D ligand)^103^ for MDA-MB-231 cells, as reported in the literature. To further validate these interactions, we explored behaviour of mimics in a complex tumour-like setting using 3D spheroids. Spheroids closely resemble in vivo conditions, fostering cell-cell interactions, nutrient gradients, and heterogeneity. Moreover, they secrete ECM, enhancing their physiological relevance for studying tumour biology and therapeutics compared to 2D cultures.^104,105^ Interaction studies were conducted on 3D spheroids of liver (HepG2) and colon (HT-29) cancer cells, maintaining similar cell-to-sphere ratios as in 2D cultures. cGCM exhibited closer proximity and higher interaction in both small and large spheroids, even at low cell to cGCM ratio (1:5) when compared to cG (Figure 6(b)). These results highlight the mimics’ capability to navigate complex matrix environments while preserving cell membrane and assembly integrity as confirmed by microscopy (Figure 6(c)). Furthermore, based on the existing literature, the presence of CD155 (DNAM-1 ligand),^106^ ULBP4-I, ULBP4-II, ULBP4-III and RAET1G3 (NKG2D ligand),^107^ and B7-H6 (NKp30 ligand)^108^ for HepG2 cells and CD155 (DNAM-1 ligand)^109,110^ and B7-H6 (NKp30 ligand)^111^ for HT-29 cells supports the observed interactions. These ligands are known to engage activating receptors on NK cells and play a critical role in NK cell-mediated immune surveillance. Since the cGCM retains the natural NK cell membrane, it is likely to display these key recognition receptors (DNAM-1, NKG2D and NKp30), enabling ligand-receptor engagement with cancer cells and promoting enhanced proximity and interaction, as observed in our in vitro and in vivo models. This complementary expression pattern between tumour ligands and NK membrane receptors provides a strong rationale for the selective targeting behaviour of cGCM. Although the current study focuses on functional targeting outcomes, receptor-level mechanisms could be investigated more specifically in future studies. Such approaches might include receptor-blocking assays using neutralizing antibodies, ligand competition studies, or gene-silencing techniques to selectively knock down NK cell membrane receptors. These strategies would enable a more precise understanding of the molecular basis behind the targeting behaviour observed here and would further delineate the specific contribution of individual receptors such as DNAM-1, NKp30, and NKG2D. Flow cytometry-based quantification of ligand binding and downstream signalling activation could also provide complementary evidence for mechanistic elucidation. Overall, these findings highlight the importance of preserving NK membrane composition during mimic fabrication and suggest that receptor-ligand recognition plays a key role in promoting tumour-targeting behaviour. The current evidence from both 2D and 3D models demonstrates the functional relevance of these interactions, laying the foundation for future molecular studies to probe the mechanistic basis of mimic–tumour cell specificity.

Although macrophage and tumour cell evaluations were conducted separately to allow for the use of optimized, cell-specific conditions and analytical tools, the resulting data collectively highlight the functional selectivity of cGCMs. The reduced uptake by macrophages, alongside increased interaction with tumour cells in both 2D and 3D models, demonstrates the dual functionality intended in NK cell mimics—simultaneous immune evasion, and tumour targeting. While the current study design offers strong foundational evidence of this selective interaction, co-culture systems incorporating both macrophages and tumour spheroids represent a valuable future direction to further explore these dynamics in a more integrated, physiologically relevant environment. Additionally, the in vivo zebrafish model used here inherently captures interactions between immune and tumour components, providing complementary insights into cellular behaviour within a live, multicellular system.

Zebrafish embryos and larvae are the second most commonly used animal models for medical, biological, and biotechnological studies. To evaluate the effectiveness of our NK cell mimics, we used a zebrafish breast cancer xenograft model, which is increasingly recognized in cancer research.^46,112^ They presents a straightforward approach for tumour development by transplantation of tumour cell lines or patient-derived tumour cells by eliminating the need for immunosuppression. Their optical transparency allows for real-time imaging and tracking of drug carriers,^34–37^ making them ideal for high-throughput screening and reducing the need for large numbers of cells and carriers compared to other animal models.^38–40^ Zebrafish larvae are particularly advantageous for studying cell-cell or cell-particle interactions in vivo, as these interactions can be observed with single-cell or even subcellular resolution over time. Given these benefits, we chose zebrafish larvae to investigate how our NK cell mimics (microspheres) interact with tumour cells and their ability to evade macrophage elimination. This approach provides unique insights that would be difficult to obtain from other animal models. The zebrafish larval model is internationally recognized for such studies, making it the optimal platform for our research.^48^ We utilized the zebrafish breast tumour (MDA-MB-231) xenograft model and applied a previously established microinjection protocol to visualize and analyse the interactions between our spheres (cG and cGCM) and tumour cells over time.^57,67,68^ The protocol was slightly modified to facilitate the microinjection of cG, owing to their positive charge. Borosilicate glass needles are inherently negatively charged and were pre-coated with Poly-L-Lysine (PLL) to reduce surface adhesion and improve ease of injection. A substantial increase in interaction between tumour cells and cGCM was evident when compared to cG (Figure 7). This underscores the superior ability of cGCM to maintain interactions with breast tumour cells over time, primarily due to their intact activating receptors (DNAM-1, NKp30, and NKG2D) and adhesion receptor (CD11a) capable of binding firmly with highly expressed ligands on MDA-MB-23.^100–103,113,114^ Next, we evaluated the stealth properties of cGCM using the Kdrl:EGFP Spil: Ds Red zebrafish model, which mimics the innate immune system during early-stage human development.^115^ The results, observed at 24 h post-injection, revealed a greater number of macrophages clustering around cG compared to cGCM (Figure 8). The uptake of cGCM was ~6.54% times lower than cG confirming their ability to evade macrophage detection post assembly and successful translation of NK cell like properties. While the microspheres developed in this study are approximately 10 µm in diameter, which may be considered large for systemic delivery, this design choice is intentional, and aligned with our goal of mimicking the size and behaviour of natural immune effector cells such as NK cells. This cell-scale dimension facilitates biomimetic functions, including cell–cell contact, tumour retention, and interaction with the tumour microenvironment. Furthermore, the intended application of these microspheres is through localized delivery routes—such as intratumoral or peritumoral injection—which are well-established in clinical oncology and compatible with larger particle sizes. Similar approaches have been successfully employed in clinically approved microsphere systems used for embolization and depot-based drug release. Therefore, while systemic administration poses size-related challenges, the current platform is designed for local application, where particle size supports both functional mimicry and translational feasibility.

Furthermore, to demonstrate the capability of cGCM to encapsulate and deliver therapeutic payloads for cancer-related applications, we conducted a pilot study targeting altered glycans, specifically by inhibiting sialyltransferases (STIs).^49^ Sialyltransferases is an enzyme that plays a crucial role in the process of glycosylation, responsible for attaching sialic acid to sugar chains on glycoproteins and glycolipids. The upregulation of sialyltransferases on cancer cells and consequently hypersialylation, contributes to several malignant properties of cancer cells, including increased cell proliferation, survival, metastasis, and evasion of the immune system. Therefore, targeting overactive sialyltransferases can be a promising strategy for drug intervention, prompting numerous investigations into effective STIs.^52,53,116^ Some of the explored STI from the literature are sialic acid analogues,^54^ CMP-sialic acid analogues,^117^ cytidine analogues,^118^ aromatic compounds^119^ etc. For our pilot study we selected 3Fax-Peracetyl Neu5Ac^54–56^ a structural analogue of sialic acid as a payload for delivery. 3Fax-Peracetyl Neu5Ac binds to the active site of sialyltransferases, downregulating their activity, and inhibiting hypersialylation. Maintaining the functionality, and precise delivery of these inhibitors is critical. To address this, a pilot study to assess the behaviour of 3Fax-Peracetyl Neu5Ac within cGCM was conducted. The release profile of STI from both cG and cGCM increased over time. Additionally, the cell membrane in NK cell mimics could further protect STI and prevent its premature release from cGCM and therefore, percentage of STI released from cG was slightly higher than from cGCM was observed. Further, to evaluate the release mechanism of 3Fax-Peracetyl Neu5Ac from NK cell mimics, we fitted the in vitro release profiles to common kinetic models, including zero-order, first-order, and Higuchi models. Among these, the zero-order model exhibited the best fit (*R^2^* = 0.93 for cGCM and 0.92 for G), indicating a near-constant release rate over time. This suggests that the release is predominantly governed by a matrix-based or surface erosion mechanism, rather than by diffusion (as in the Higuchi model) or concentration-dependent first-order kinetics. The limited fit to first-order and Higuchi models further supports this interpretation. Given that surface erosion maintains a relatively stable release rate, this feature may be advantageous for glycan modulation therapies, where prolonged, predictable dosing is essential for therapeutic effect. Although a direct in vivo correlation to therapeutic efficacy was beyond the scope of this study, our results lay the foundation for future exploration of the immune-modulating potential of STI-loaded NK cell mimics in cancer therapy. To investigate the sialylation status and glycan alterations on the surface of MDA-MB-231 cells following treatment with sialyltransferase inhibitor (STI, 3Fax-Peracetyl Neu5Ac)-loaded carriers, we employed a panel of fluorescently labelled lectins—SNA, PNA, WGA, and MAA—each chosen for its specific glycan-binding affinity. SNA (Sambucus nigra agglutinin) selectively binds to α2,6-linked sialic acid residues, a terminal glycan modification frequently upregulated in cancer and associated with immune evasion and enhanced tumour cell survival. A significant reduction in SNA fluorescence intensity was observed in cells treated with STI, cG_STI, and cGCM_STI, indicating that the delivery of STI via gelatin microspheres effectively inhibited α2,6-sialylation (Figure 9(b) and (c)). This observation is consistent with the known specificity of 3Fax-Peracetyl Neu5Ac (STI) for α2,6-sialyltransferases and supports the efficacy of the delivery system in modulating tumour-associated glycosylation. In contrast, no significant changes were observed in the fluorescence intensities of MAA, WGA, and PNA lectins following treatment (Figure 9(d)–(f)). MAA (Maackia amurensis agglutinin) binds to α2,3-linked sialic acids, which are likely regulated by different sialyltransferases not targeted by STI. The absence of change in MAA binding thus reinforces the specificity of STI for α2,6 linkages. WGA (wheat germ agglutinin), which has broader binding specificity for both sialic acid and N-acetylglucosamine residues, also showed no significant variation in fluorescence. This suggests that global glycan expression or total sialic acid content was not markedly altered, further indicating that STI selectively affects α2,6-linked structures rather than causing widespread desialylation. Likewise, PNA (peanut agglutinin), which binds to the Galβ1-3GalNAc disaccharide typically masked by sialic acids, showed unchanged binding, implying that desialylation did not unmask this motif. This again supports the conclusion that STI selectively inhibits α2,6-sialyltransferases and does not broadly disrupt the sialylation landscape. Together, these findings highlight the selective action of STI on α2,6-linked sialic acids and demonstrate the value of multi-lectin staining to map glycan alterations in response to targeted glycosylation-modulating therapies.

Nonetheless, STI-cGCM retained functional integrity of the inhibitor, as evidenced by a significant reduction in α-2,6 sialylation on MDA-MB-231 cells, confirming that the membrane coating did not compromise therapeutic activity. These findings not only validate the potential of cGCMs for targeted therapeutic delivery but also illustrate their ability to maintain payload bioactivity while offering additional layers of biological functionality, such as immune evasion and tumour targeting. While this was a preliminary investigation, it establishes a foundation for future studies exploring STI-loaded NK cell mimics in the context of tumour glycan modulation and precision cancer therapy. It is also important to highlight that the interaction and sialylation studies were performed without incorporating cytotoxic payloads; thus, the main objective was to assess NK cell mimic–tumour cell interactions and the controlled release of functional glycan modulators. These foundational findings pave the way for future designs combining sialylation-targeting strategies with chemotherapeutic agents for enhanced tumour cell killing.

## Conclusion

In conclusion, we developed a safe and effective method for cross-linking gelatin microspheres using the non-toxic cross-linker DMTMM, enhancing their stability with tunable mechanical properties. These modified microspheres were successfully coated with KHYG-1 cell membranes, creating NK cell mimics with surface characteristics, size, and relevant cell-like mechanical properties. These NK cell mimics demonstrated tissue and cell compatibility, reduced macrophage uptake, and did not elicit any pro-inflammatory response. In vitro studies confirmed their enhanced interaction with tumour cells, even at low concentrations in both 2D and 3D tumour cell cultures. Moreover, NK cell mimics showed enhanced tumour interaction in zebrafish breast tumour xenograft model and can elude macrophage detection. Additionally, the loading and release of a sialyltransferase inhibitor (STI) from these mimics were effective and reduces sialylation on breast tumour cells suggesting potential therapeutic applications. These findings collectively highlight the promising potential of NK cell mimics for targeted cancer therapies.

## Supplemental Material

sj-docx-1-tej-10.1177_20417314251349675 – Supplemental material for Design of an artificial natural killer cell mimicking system to target tumour cells Supplemental material, sj-docx-1-tej-10.1177_20417314251349675 for Design of an artificial natural killer cell mimicking system to target tumour cells by Vaishali Chugh, Vijaya Krishna Kanala, Dagmar Quandt, Suainibhe Kelly, Damien King, Lasse D. Jensen, Jeremy C. Simpson and Abhay Pandit in Journal of Tissue Engineering

sj-tif-10-tej-10.1177_20417314251349675 – Supplemental material for Design of an artificial natural killer cell mimicking system to target tumour cells Supplemental material, sj-tif-10-tej-10.1177_20417314251349675 for Design of an artificial natural killer cell mimicking system to target tumour cells by Vaishali Chugh, Vijaya Krishna Kanala, Dagmar Quandt, Suainibhe Kelly, Damien King, Lasse D. Jensen, Jeremy C. Simpson and Abhay Pandit in Journal of Tissue Engineering

sj-tif-11-tej-10.1177_20417314251349675 – Supplemental material for Design of an artificial natural killer cell mimicking system to target tumour cells Supplemental material, sj-tif-11-tej-10.1177_20417314251349675 for Design of an artificial natural killer cell mimicking system to target tumour cells by Vaishali Chugh, Vijaya Krishna Kanala, Dagmar Quandt, Suainibhe Kelly, Damien King, Lasse D. Jensen, Jeremy C. Simpson and Abhay Pandit in Journal of Tissue Engineering

sj-tif-2-tej-10.1177_20417314251349675 – Supplemental material for Design of an artificial natural killer cell mimicking system to target tumour cells Supplemental material, sj-tif-2-tej-10.1177_20417314251349675 for Design of an artificial natural killer cell mimicking system to target tumour cells by Vaishali Chugh, Vijaya Krishna Kanala, Dagmar Quandt, Suainibhe Kelly, Damien King, Lasse D. Jensen, Jeremy C. Simpson and Abhay Pandit in Journal of Tissue Engineering

sj-tif-3-tej-10.1177_20417314251349675 – Supplemental material for Design of an artificial natural killer cell mimicking system to target tumour cells Supplemental material, sj-tif-3-tej-10.1177_20417314251349675 for Design of an artificial natural killer cell mimicking system to target tumour cells by Vaishali Chugh, Vijaya Krishna Kanala, Dagmar Quandt, Suainibhe Kelly, Damien King, Lasse D. Jensen, Jeremy C. Simpson and Abhay Pandit in Journal of Tissue Engineering

sj-tif-4-tej-10.1177_20417314251349675 – Supplemental material for Design of an artificial natural killer cell mimicking system to target tumour cells Supplemental material, sj-tif-4-tej-10.1177_20417314251349675 for Design of an artificial natural killer cell mimicking system to target tumour cells by Vaishali Chugh, Vijaya Krishna Kanala, Dagmar Quandt, Suainibhe Kelly, Damien King, Lasse D. Jensen, Jeremy C. Simpson and Abhay Pandit in Journal of Tissue Engineering

sj-tif-5-tej-10.1177_20417314251349675 – Supplemental material for Design of an artificial natural killer cell mimicking system to target tumour cells Supplemental material, sj-tif-5-tej-10.1177_20417314251349675 for Design of an artificial natural killer cell mimicking system to target tumour cells by Vaishali Chugh, Vijaya Krishna Kanala, Dagmar Quandt, Suainibhe Kelly, Damien King, Lasse D. Jensen, Jeremy C. Simpson and Abhay Pandit in Journal of Tissue Engineering

sj-tif-6-tej-10.1177_20417314251349675 – Supplemental material for Design of an artificial natural killer cell mimicking system to target tumour cells Supplemental material, sj-tif-6-tej-10.1177_20417314251349675 for Design of an artificial natural killer cell mimicking system to target tumour cells by Vaishali Chugh, Vijaya Krishna Kanala, Dagmar Quandt, Suainibhe Kelly, Damien King, Lasse D. Jensen, Jeremy C. Simpson and Abhay Pandit in Journal of Tissue Engineering

sj-tif-7-tej-10.1177_20417314251349675 – Supplemental material for Design of an artificial natural killer cell mimicking system to target tumour cells Supplemental material, sj-tif-7-tej-10.1177_20417314251349675 for Design of an artificial natural killer cell mimicking system to target tumour cells by Vaishali Chugh, Vijaya Krishna Kanala, Dagmar Quandt, Suainibhe Kelly, Damien King, Lasse D. Jensen, Jeremy C. Simpson and Abhay Pandit in Journal of Tissue Engineering

sj-tif-8-tej-10.1177_20417314251349675 – Supplemental material for Design of an artificial natural killer cell mimicking system to target tumour cells Supplemental material, sj-tif-8-tej-10.1177_20417314251349675 for Design of an artificial natural killer cell mimicking system to target tumour cells by Vaishali Chugh, Vijaya Krishna Kanala, Dagmar Quandt, Suainibhe Kelly, Damien King, Lasse D. Jensen, Jeremy C. Simpson and Abhay Pandit in Journal of Tissue Engineering

sj-tif-9-tej-10.1177_20417314251349675 – Supplemental material for Design of an artificial natural killer cell mimicking system to target tumour cells Supplemental material, sj-tif-9-tej-10.1177_20417314251349675 for Design of an artificial natural killer cell mimicking system to target tumour cells by Vaishali Chugh, Vijaya Krishna Kanala, Dagmar Quandt, Suainibhe Kelly, Damien King, Lasse D. Jensen, Jeremy C. Simpson and Abhay Pandit in Journal of Tissue Engineering
