# Supplementary material for: Design of an artificial natural killer cell mimicking system to target tumour cells
Source: J Tissue Eng. 2025 Sep 27;16:20417314251349675. doi: 10.1177/20417314251349675 (PMC12477366; doi:10.1177/20417314251349675)
Supplement: sj-docx-1-tej-10.1177_20417314251349675 – Supplemental material for Design of an artificial natural killer cell mimicking system to target tumour cells [file sj-docx-1-tej-10.1177_20417314251349675.docx]

**Supplementary information**

**Design of an Artificial Natural Killer Cell Mimicking System to Target Tumour Cells**

Vaishali Chugh^a*^, K. Vijaya Krishna^a^, Dagmar Quandt^a^, Suainibhe Kelly^b^, Damien King^c^, Lasse D. Jensen^d^, Jeremy C Simpson^b^, Abhay Pandit^a*^

^a^CÚRAM, SFI Research Centre for Medical Devices, University of Galway, Galway, H92W2TY, Ireland

^b^Cell Screening Laboratory, UCD School of Biology and Environmental Science, University College Dublin, Belfield, Dublin 4, Ireland

^c^Fraunhofer Project Centre for Embedded BioAnalytical Systems, Dublin City University, Dublin 9, Ireland

^d^Department of Health, Medicine and Caring Sciences, Linköping University, 58183, Linköping, Sweden

*Corresponding author: [vaishalichugh04@gmail.com](mailto:vaishalichugh04@gmail.com), [abhay.pandit@universityofgalway.ie](mailto:abhay.pandit@universityofgalway.ie)

1. **Materials and Methods**
   1. *Determination of surface charge and particle size distribution*

The determination of particle size distribution was done in Malvern Mastersizer 2000 laser diffraction particle size analyser (with Scirocco 2000 and Hydro 2000 µP accessories) by dry method. Around 0.5 mg was analysed in triplicates in the range of 0.01 μm to 3500 μm. The surface charge on the microspheres were determined using Malvern Instruments ZEN2600 Zetasizer Nano. Concentration of 0.5 mg/ml gelatin microspheres and NK cell mimics in deionized water was sonicated in water bath sonicator (5 min) and analysed in triplicates in zetasizer.

- 1. *Fourier Transform Infrared (FT-IR) study*

FT-IR spectra was obtained on an IR spectrophotometer with a deuterated triglycine sulfate (DTGS). Before all the measurements, all the samples (cross-linked gelatin microspheres, isolated cell membrane and NK cell mimics) were freeze dried. The scanning range was set from 400-4000 cm^-1^.

- 1. *Determination of extent of crosslinking*

The percentage of crosslinking or free amino groups in the cross-linked gelatin microspheres (N=3) was determined using 2,4,6-Trinitrobenzene Sulfonic Acid (TNBS) assay.[[1]] 5 mg of cross-linked gelatin microspheres were taken in 15 ml of falcon tube. First, 1 ml of sodium bicarbonate (NaHCO_3_, 4% w/v) was added and then 1 ml of TNBS (0.5% w/v) solution in deionized water. Further, kept the reaction at 40 °C for 2 h. After 2 h, 3 ml of 6 M hydrochloric acid (HCl) was added and temperature was raised to 60 °C for 90 min to solubilize the gelatin microspheres. At last, 5 ml of deionised water was added in the tube. The resulting solution was analysed in triplicates and measured the absorbance at 345 nm using plate reader spectrometer. A control (non-cross-linked gelatin microspheres) was prepared with the same procedure except the HCl was added before the addition of TNBS. For the standard curve, different concentration of gelatin (2-10 mg/ml) was used.

- 1. *In vitro cell metabolic activity:* AlamarBlue® assay

AlamarBlue® assay was used to assess the cells metabolic activity in the presence of the compounds. Briefly, 50,000 THP-1 cells were seeded in 24 well plate and differentiated using 100 ng/ml phorbol 12-myristate 13-acetate for 24 h. After PMA treatment, THP-1 cells adhered to the surface of the well plate. Further, removed the old medium gently and washed cells with dulbecco's phosphate-buffered saline (DPBS) twice. Briefly, different concentration (10, 25, 50 and 100 μg/ml) of gelatin microspheres (cG) and NK cell mimics (cGCM) prepared in THP-1 culture medium were added with the differentiated cells in each well. For positive control, differentiated THP-1 cells were treated with its culture media for 24 h. Each treatment was done in triplicates. After 24 h treatment of spheres, the media and the spheres were removed, and a 10% solution of alamarBlue in phosphate-buffered saline (PBS) was put in contact with the cells for 3 h. Afterwards, the solution was removed and the absorbance read at 450 nm and 550 nm using a Varioskan Flash plate reader (ThermoFisher) was done.

- 1. *Calculation of Macrophage Interaction with Gelatin Microspheres and NK Cell Mimics*

The co-localization of macrophages with gelatin microspheres (cG) and NK cell mimics (cGCM) was quantified by measuring the yellow co-localized regions in fluorescence images, where macrophages (labelled with red fluorescence) interacted with FITC-labeled microspheres. The calculation of macrophage interaction was performed using the following formula:

$$Macrophage interaction or uptale \left( \% \right)=\frac{Area of interacted gelatin microspheres or NK cell mimics \left( yellow co-localization \right)}{Area of total gelatin microspheres or NK cell mimics}*100$$

This formula calculates the percentage of the microsphere area that co-localized with macrophages, represented by the yellow overlap in the fluorescence images. The total area of the microspheres (green) and the yellow co-localized regions were measured using ImageJ software.

For the representative images shown in Figure 8 in the main manuscript,

• For cG microspheres, area of co-localization (yellow) was 931 µm² and Area of total cG was 3457 µm², therefore using the formula mentioned above,

931/3457× 100 = 26.93%

• For cGCM mimics, area of co-localization (yellow) was 789 µm² and Area of total cGCM was 4627 µm², therefore using the formula mentioned above,

789 / 4627 × 100 = 17.05%

This calculation provides a quantifiable measure of macrophage uptake and interaction with the microspheres, which is important for evaluating their potential therapeutic effect in vivo.

| **Primary Antibody** | **Cat. no./ Type** | **Molecular weight (kDa)** | **% SDS Gel** | **Primary Antibody Dilution** | **Secondary Antibody/ Cat. no.** | **Secondary Antibody Dilution** |
| --- | --- | --- | --- | --- | --- | --- |
| Anti-NKp30 | ab186425/Rabbit monoclonal | ~22 | 12 | 1:2000 | HRP- goat anti-rabbit/ 31460 | 1:10,000 |
| Anti-CD226  (DNAM-1) | ab128277/  Rabbit polyclonal | ~40KDa | 12 | 1:1000 | HRP- goat anti-rabbit/ 31460 | 1:10,000 |
| Anti-NKG2D | ab96606/Rabbit polyclonal | ~35 | 12 | 1:500 | HRP- goat anti-rabbit/ 31460 | 1:10,000 |
| Anti-CD11a  (LFA-1) | ab52895/Rabbit monoclonal | ~180 | 10 | 1:5000 | HRP- goat anti-rabbit/ 31460 | 1:10,000 |
| Anti-CD56 | **MA-106801****Mouse monoclonal** | ~150 | 10 | 1:1000 | HRP-goat anti-mouse/ 31460 | 1:4000 |

**Table S1.** List and parameters of primary and secondary antibodies used for western blotting application

**References**

[1] F. Graziola, T.M. Candido, C.A.d. Oliveira, D.D.A. Peres, M.G. Issa, J. Mota, C. Rosado, V.O. Consiglieri, T.M. Kaneko, M.V.R. Velasco, A.R. Baby, Gelatin-based microspheres crosslinked with glutaraldehyde and rutin oriented to cosmetics, Braz. J. Pharm. Sci 52(4) (2016) 603-612.
