# Supplementary figures and images for: Design of an artificial natural killer cell mimicking system to target tumour cells
Source: J Tissue Eng. 2025 Sep 27;16:20417314251349675. doi: 10.1177/20417314251349675 (PMC12477366; doi:10.1177/20417314251349675)

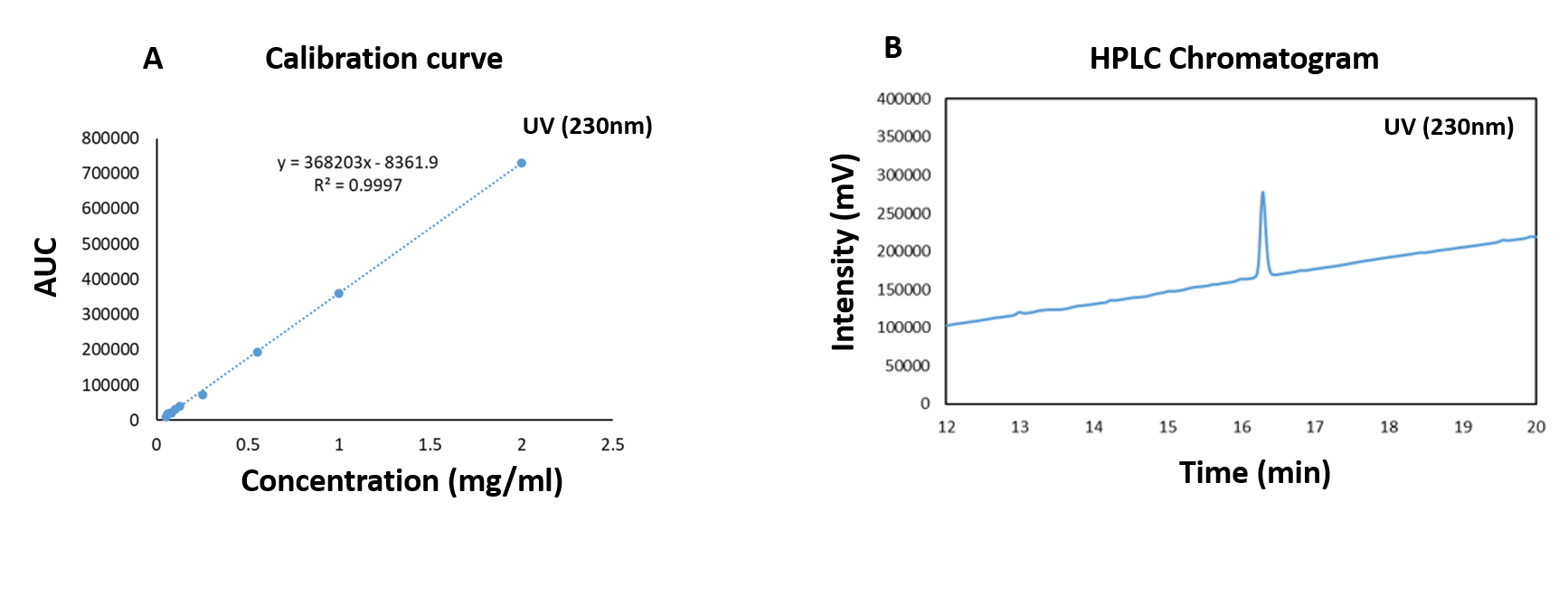

Supplement: sj-tif-10-tej-10.1177_20417314251349675 – Supplemental material for Design of an artificial natural killer cell mimicking system to target tumour cells [file sj-tif-10-tej-10.1177_20417314251349675.tif]

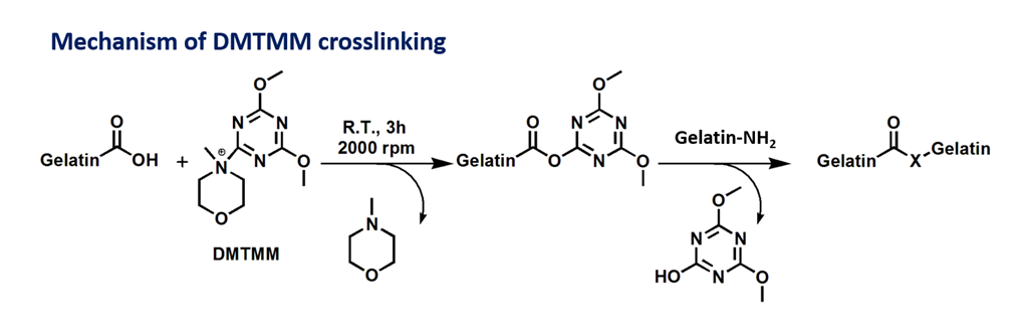

Supplement: sj-tif-11-tej-10.1177_20417314251349675 – Supplemental material for Design of an artificial natural killer cell mimicking system to target tumour cells [file sj-tif-11-tej-10.1177_20417314251349675.tif]

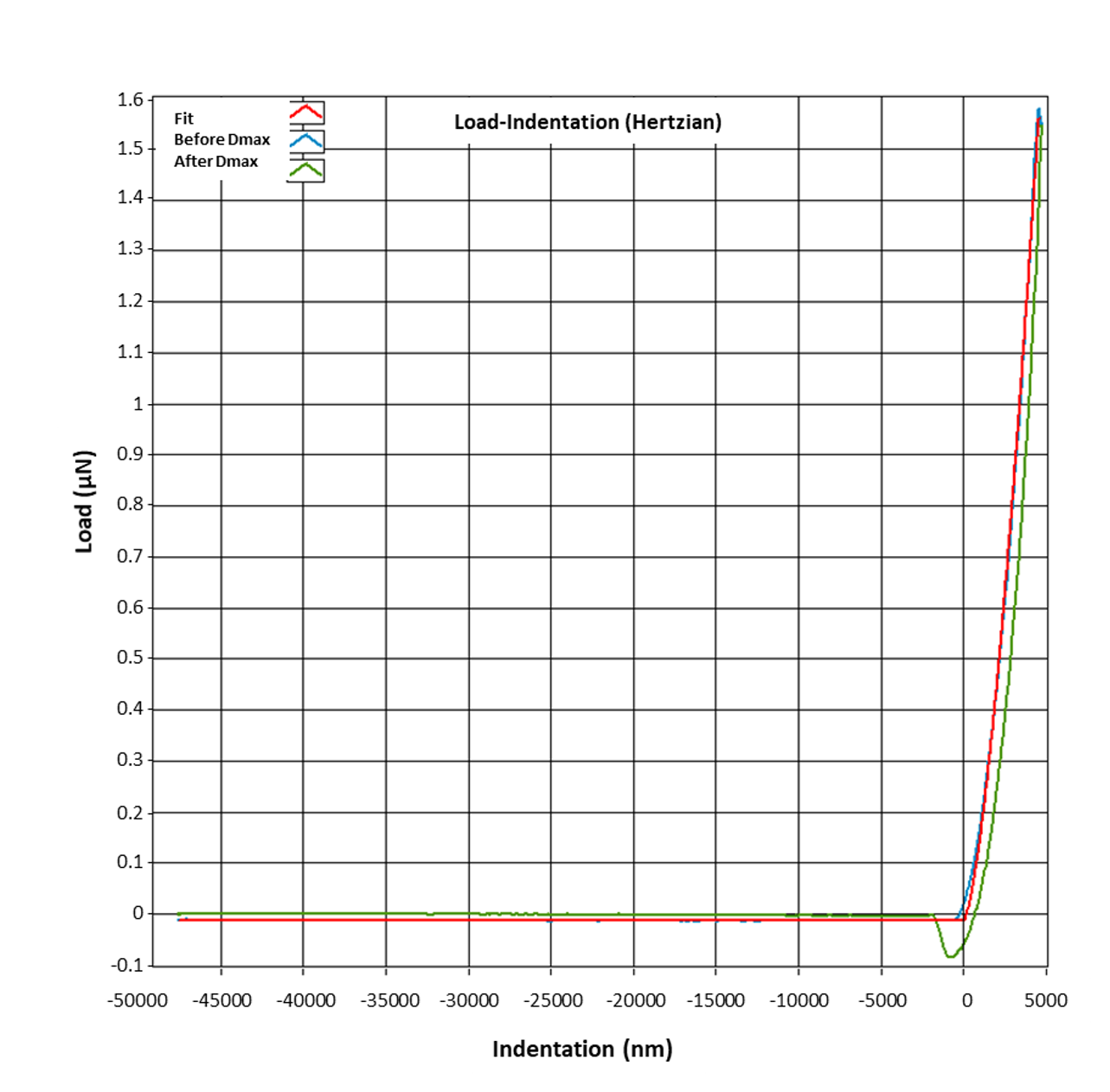

Supplement: sj-tif-2-tej-10.1177_20417314251349675 – Supplemental material for Design of an artificial natural killer cell mimicking system to target tumour cells [file sj-tif-2-tej-10.1177_20417314251349675.tif]

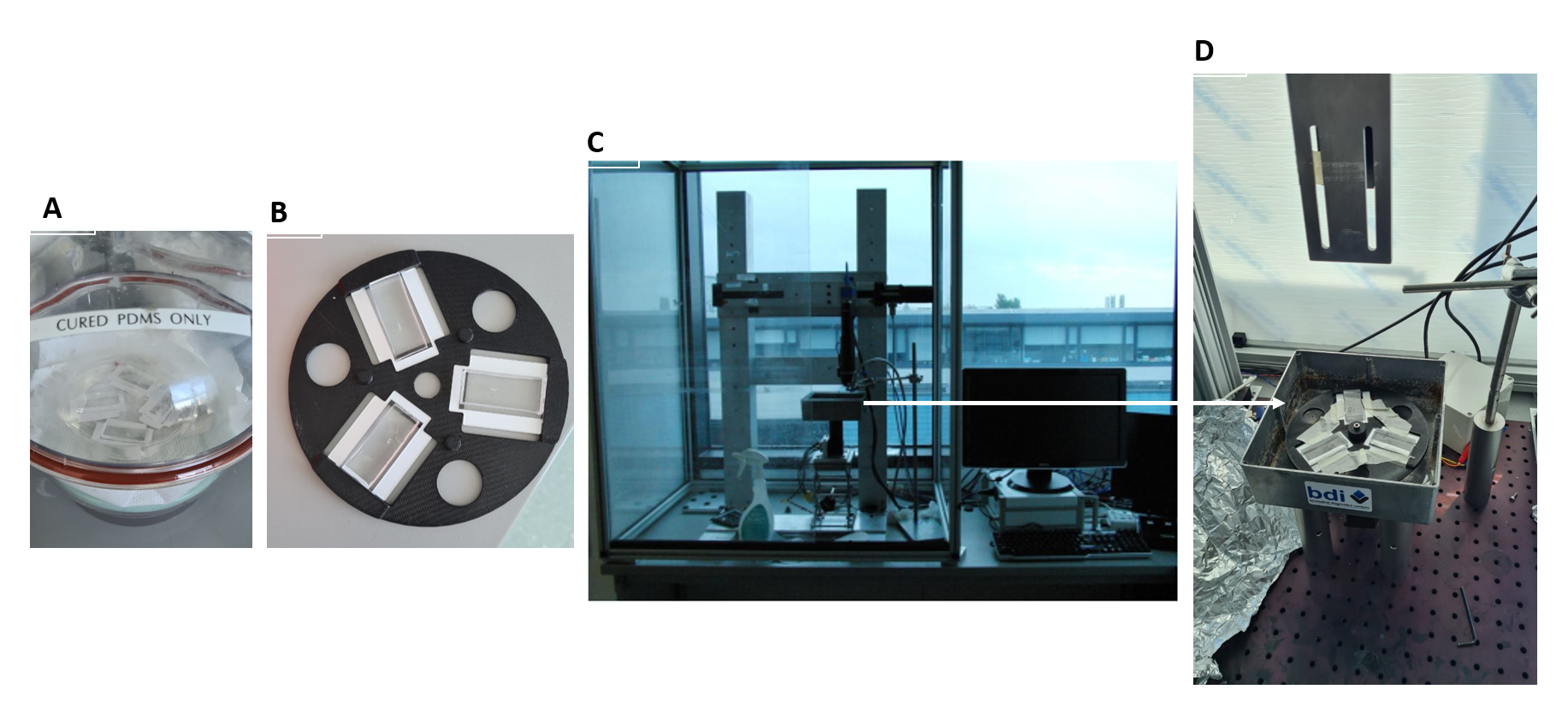

Supplement: sj-tif-3-tej-10.1177_20417314251349675 – Supplemental material for Design of an artificial natural killer cell mimicking system to target tumour cells [file sj-tif-3-tej-10.1177_20417314251349675.tif]

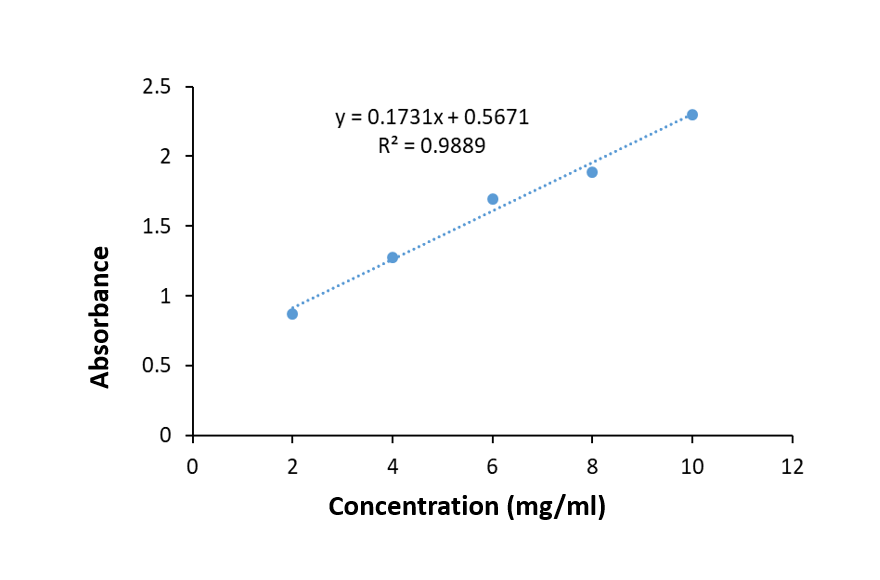

Supplement: sj-tif-4-tej-10.1177_20417314251349675 – Supplemental material for Design of an artificial natural killer cell mimicking system to target tumour cells [file sj-tif-4-tej-10.1177_20417314251349675.tif]

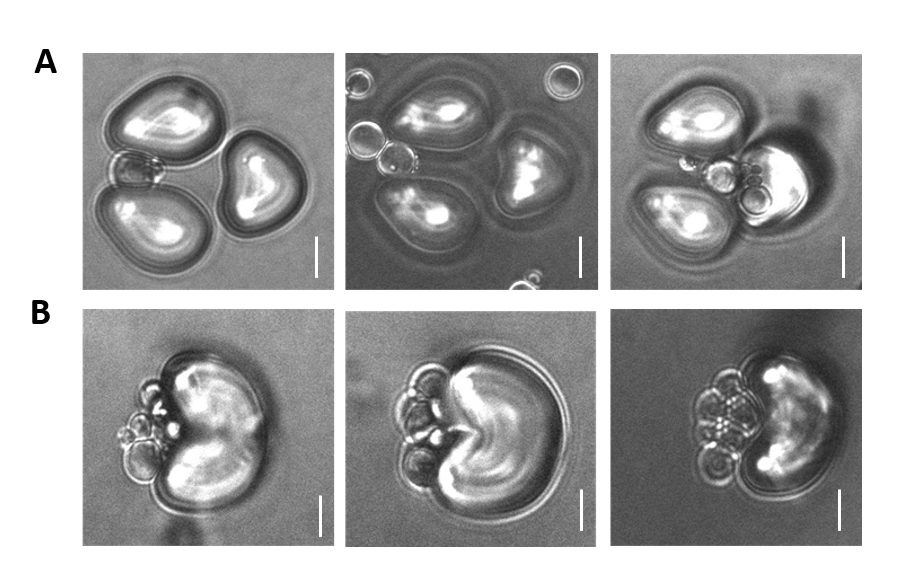

Supplement: sj-tif-5-tej-10.1177_20417314251349675 – Supplemental material for Design of an artificial natural killer cell mimicking system to target tumour cells [file sj-tif-5-tej-10.1177_20417314251349675.tif]

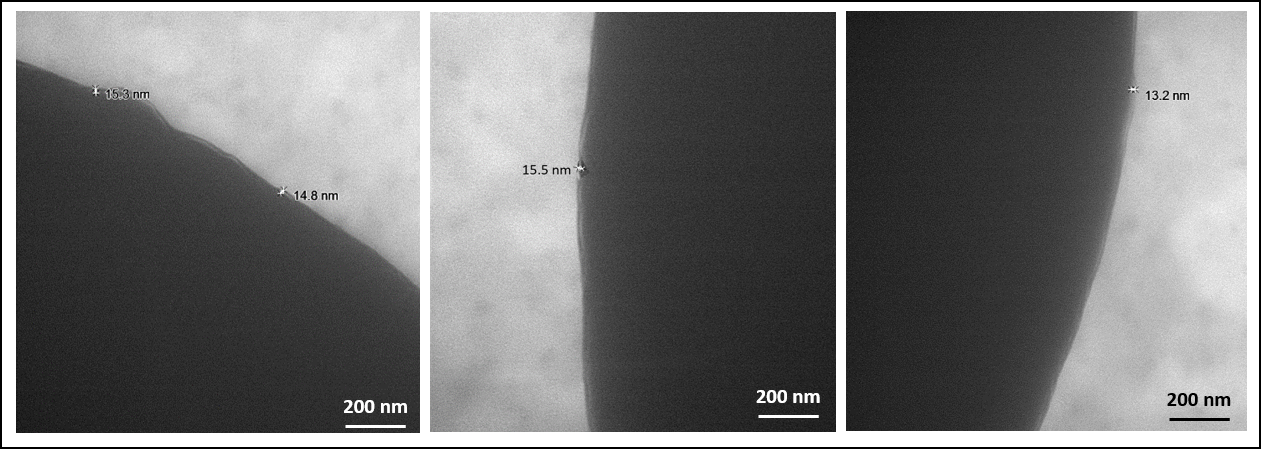

Supplement: sj-tif-6-tej-10.1177_20417314251349675 – Supplemental material for Design of an artificial natural killer cell mimicking system to target tumour cells [file sj-tif-6-tej-10.1177_20417314251349675.tif]

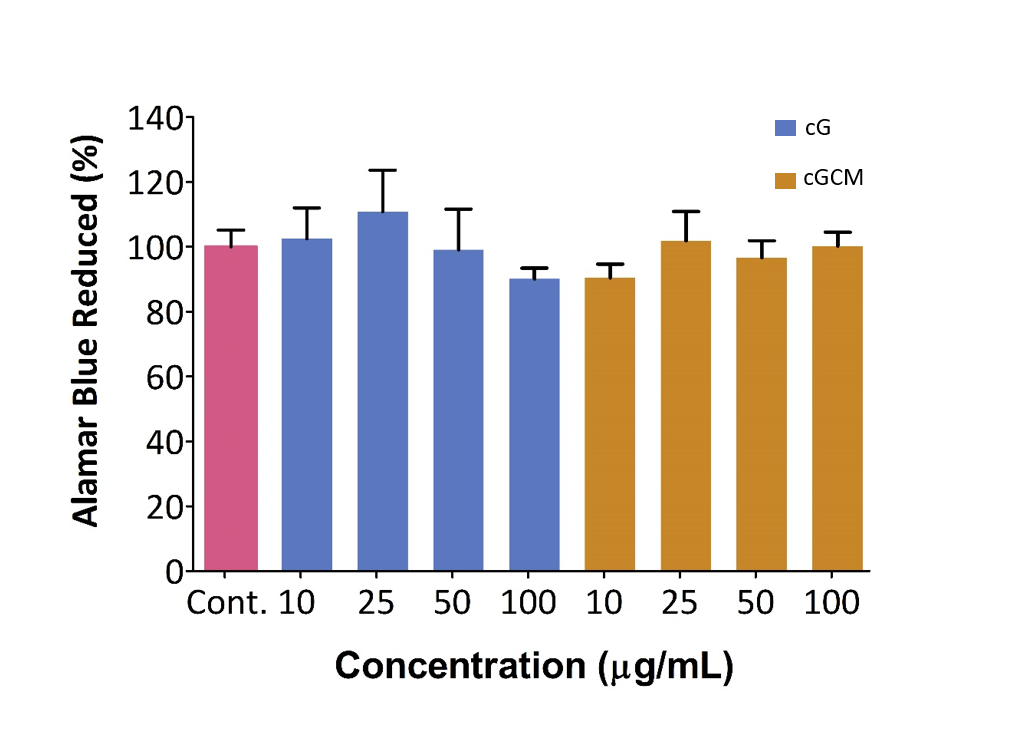

Supplement: sj-tif-7-tej-10.1177_20417314251349675 – Supplemental material for Design of an artificial natural killer cell mimicking system to target tumour cells [file sj-tif-7-tej-10.1177_20417314251349675.tif]

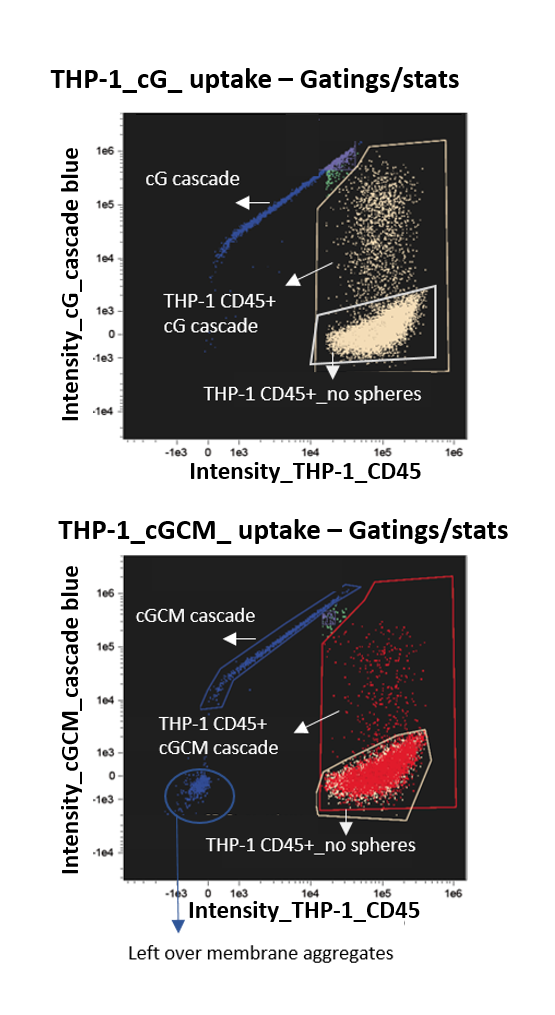

Supplement: sj-tif-8-tej-10.1177_20417314251349675 – Supplemental material for Design of an artificial natural killer cell mimicking system to target tumour cells [file sj-tif-8-tej-10.1177_20417314251349675.tif]

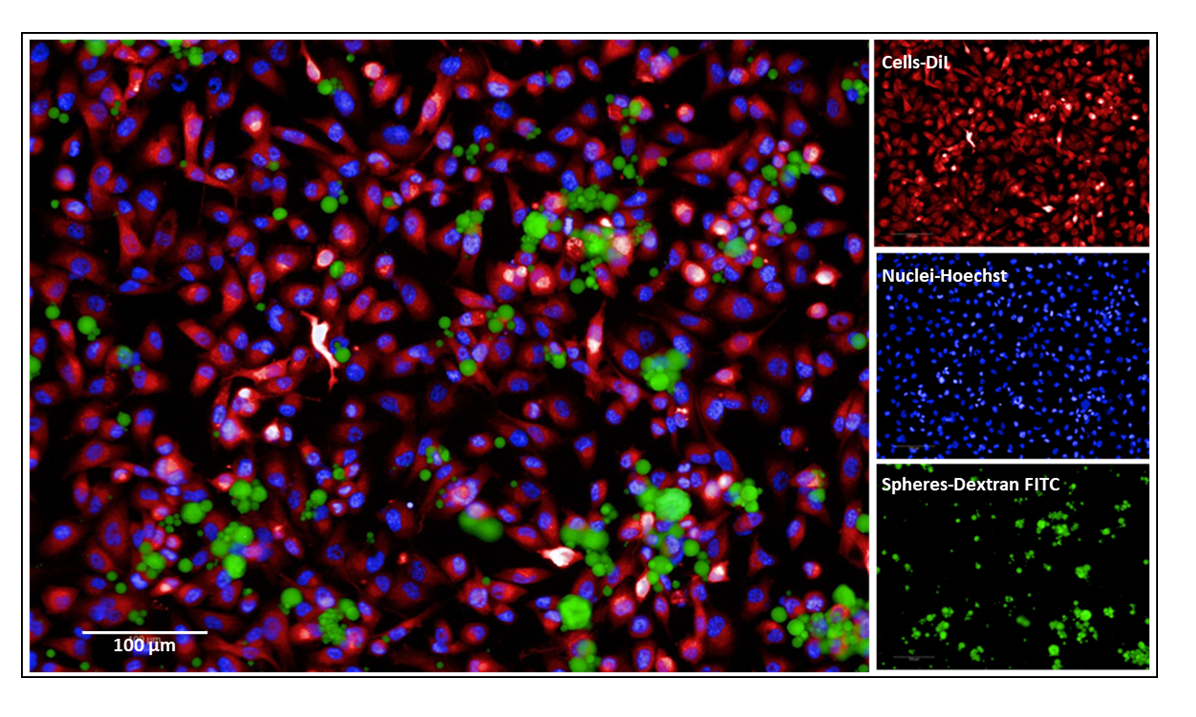

Supplement: sj-tif-9-tej-10.1177_20417314251349675 – Supplemental material for Design of an artificial natural killer cell mimicking system to target tumour cells [file sj-tif-9-tej-10.1177_20417314251349675.tif]
